# Association between gut microbiome profiles and host metabolic health across the life course: a population-based study

**DOI:** 10.1016/j.lanepe.2024.101195

**Published:** 2024-12-28

**Authors:** Ruolin Li, Alexander Kurilshikov, Shuyue Yang, Julie A.E. van Oortmerssen, Arno van Hilten, Fariba Ahmadizar, Gennady Roshchupkin, Robert Kraaij, Liesbeth Duijts, Jingyuan Fu, M. Kamran Ikram, Vincent W.V. Jaddoe, André G. Uitterlinden, Fernando Rivadeneira, Maryam Kavousi, Alexandra Zhernakova, Carolina Medina-Gomez

**Affiliations:** a Department of Internal Medicine, Erasmus MC, University Medical Center Rotterdam, Rotterdam, the Netherlands; b The Generation R Study Group, Erasmus MC, University Medical Center Rotterdam, Rotterdam, the Netherlands; c Department of Genetics, University Medical Center Groningen, Groningen, the Netherlands; d Department of Epidemiology, Erasmus MC, University Medical Center Rotterdam, Rotterdam, the Netherlands; e Department of Medical Informatics, Erasmus MC, University Medical Center Rotterdam, Rotterdam, the Netherlands; f Department of Radiology and Nuclear Medicine, Erasmus MC, University Medical Center Rotterdam, Rotterdam, the Netherlands; g Department of Data Science & Biostatistics, Julius Global Health, University Medical Center Utrecht, Utrecht, the Netherlands; h Department of Pediatrics, Erasmus MC, University Medical Center Rotterdam, Rotterdam, the Netherlands; i Department of Neurology, Erasmus MC, University Medical Center Rotterdam, Rotterdam, the Netherlands; j Department of Epidemiology, Harvard T.H. Chan School of Public Health, Boston, USA

**Keywords:** Gut microbiome, Metabolic health, Life course epidemiology, ASCVD, Observational studies, Population-based cohorts

## Abstract

**Background:**

The human gut microbiome changes considerably over time. Previous studies have shown that gut microbiome profiles correlate with multiple metabolic traits. As disease development is likely a lifelong process, evidence gathered at different life stages would help gain a better understanding of this correlation. Therefore, we aim to investigate how the association of the gut microbiome and metabolic traits change over the lifespan.

**Methods:**

We identified microbiome patterns (clusters) within two population-based cohorts at different life stages, i.e., pre-adolescents of the Generation R Study (mean age 9.8 years; n = 1488) and older adults of the Rotterdam Study (RS, mean age 62.7 years; n = 1265) using K-Means clustering, and surveyed for host metabolic phenotypes, lifestyles and other factors driving these patterns. Analyses were replicated in the Lifelines-DEEP Study (mean age 45.0 years; n = 1117). The association between microbiome clusters and host metabolic health was evaluated as well as the link between microbiome clusters and incident atherosclerotic cardiovascular disease (ASCVD) in RS during follow-up (median 6.5 years).

**Findings:**

We identified two distinct microbiome clusters (U and H) within each study population presenting contrasting metabolic statuses. Cluster U was characterized by lower microbiome diversity, increased *Streptococcus*, *Fusicatenibacter*, and decreased *Prevotella_9* and *Christensenellaceae_R-7_group*; wherein individuals showed higher fat percentage, triglycerides, use of medications, and lower socioeconomic status. Individuals in cluster U had increased odds (between 1.10 and 1.65) of being relatively metabolically unhealthy and presented a higher 5-year ASCVD risk (mean risk 0.059 ± 0.071 vs 0.047 ± 0.042, p < 0.001).

**Interpretation:**

We provide evidence of a life–course relationship between gut microbiome profiles and metabolic health.

**Funding:**

R.L is supported by 10.13039/501100000780European Union10.13039/501100007601Horizon 2020 research and innovation program under Marie Skłodowska-Curie grant agreement No 860898 [FIDELIO].

**Research in context**

**Evidence before this study**

Previous evidence for the association between gut microbiome profiles and metabolic health status is largely based on selected individuals from a clinical setting, usually scrutinizing a small fraction of the population presenting with a specific health condition. We searched PubMed for peer-reviewed papers published from inception to 01/07/2024 using the searching terms as follows: ((gut microbio∗[MeSH Terms]) OR (intestinal microbio∗[MeSH Terms])) OR ((metabolic disease∗[MeSH Terms]) OR (metabolic health [MeSH Terms])) AND (population [MeSH Terms]) AND ((life-course [MeSH Terms]) OR (lifespan [MeSH Terms])). Despite the many studies evaluating metabolic traits and gut microbiome profiles, to our knowledge, there are no studies that investigated lifelong age-related changes in the relation of microbiome profiles and metabolic health in the general population.

**Added value of this study**

We assessed the data of 3870 participants from three deeply-phenotyped Dutch cohorts comprising different life stages (from childhood to late adulthood). Our study established that gut microbiome profiles correlate with overall metabolic health status in the general population across different life stages; yet, such an association is stronger in the elderly than in the pre-adolescents. Furthermore, this study provides evidence that in older adults, presenting an unhealthy gut microbiome pattern is indicative of an increased 5-year risk of incident atherosclerotic cardiovascular disease (ASCVD).

**Implications of all the available evidence**

Our study revealed that the gut microbiome is reflective of the overall metabolic health status, both in children and older adults. Lower socioeconomic status, higher body fat percentage, higher blood triglycerides, and obesity are associated with an unhealthy gut microbiome pattern (characterized by a less-diverse microbiome and dissimilar taxonomic composition) in all age groups. More specifically, our study implicated the childhood and adolescence period as a suitable lifetime window for the prevention of metabolic disorders across the whole lifespan.

## Introduction

Metabolic disorders such as obesity, type 2 diabetes mellitus (T2D), and metabolic syndrome in adults are increasingly prevalent. The rising burden of metabolic diseases is a major concern in healthcare worldwide, whereas the origin of many metabolic diseases can be traced back to the childhood period.^1^ Globally, about 3% of children and 5% of adolescents were affected with metabolic syndrome in 2020.^2^ Childhood and adolescence are critical periods in which growth and development are transformative and can deeply affect health later in life; therefore, metabolic health during this period could be a key determinant of metabolic diseases in adulthood.

The gut microbiome, commonly assessed in stool, comprises a collection of microorganisms harbored in the digestive tract that impact human physiology through different biological processes. Microbial community compositions vary over the lifespan. At birth, the taxonomic diversity is relatively low but increases rapidly as the infant is colonized with bacteria acquired mainly from breast milk and the environment.^3^ In childhood, the gut microbiome is not completely mature and represents a dynamic community, likely influencing health outcomes later in life. Pubertal changes in hormone levels dominate shifts in the microbial communities during adolescence which exhibit a particular signature.^4^ In contrast, the gut microbiome remains relatively stable through adulthood, except following perturbations such as infections, antibiotics or drastic dietary interventions. In elderly years, there is a decline in diversity starting around 65 years and becoming more pronounced in individuals older than 80 years, during these years also a depletion of known healthy microbial communities is evidenced.^3^

Previous reports have indicated a link between altered gut microbiome profiles and human metabolic health status.^5^ However, many studies present inconsistent findings likely due to limitations such as small or highly selected study populations, differences in the collection and processing of samples, and statistical modeling of the data.^6^ Thus, it is still unclear whether the reported microbiome associations are relevant to the general population and if they change over the lifespan.

Therefore, we investigated the life–course relationship between gut microbiome and broad host metabolic profiles in population-based cohorts. We hypothesized that microbiome metrics such as diversity and gain or loss of specific taxa are associated with health status in children and adults even after considering sociodemographic and lifestyle factors. We used data from three Dutch cohorts representing different age stages and followed a microbiome-wide approach focused on phenotypes mainly comprising components of the metabolic syndrome. Our purpose was to establish different gut microbiome patterns in the general population, and to identify the specific gut microbiome features driving such a clustering. In addition, given the high burden of cardiovascular diseases, we evaluated the association of the identified gut microbiome patterns with incident atherosclerotic cardiovascular disease (ASCVD) in old adults.

## Methods

### Study populations

This study includes three Dutch population-based studies. Among the three cohorts, the Generation R Study and the Rotterdam Study were conducted by Erasmus MC, University Medical Center Rotterdam in Rotterdam, the Netherlands; the Lifelines-DEEP Study was conducted by the University Medical Centre Groningen in Groningen, the Netherlands.

The Generation R Study (GenR) is a population-based prospective multi-ethnic birth cohort conducted in Rotterdam and aims to identify early environmental and genetic factors underlying growth, development, and health before adulthood.^7^ In total, stools from 2921 participants (mean age 10 years) were collected.^8^ The Rotterdam Study (RS) is a prospective population-based cohort study established in 1990 in Ommord, a suburb of Rotterdam, to study determinants of disease and disability in Dutch adult individuals.^9^ The fecal collection comprised 1691 individuals (mean age 63 years) of the third cohort (RS-III).^8^ All subjects provided written consent before participation in the study. The studies complied with the Declaration of Helsinki. Ethical approval was obtained from the Medical Ethical Committee of Erasmus MC (MEC-02-1015/MEC-2012-165).

The Lifelines-DEEP (LLD) Study is embedded in Lifelines, a population cohort of over 165,000 participants that covers multiple generations and focuses on determinants for multifactorial diseases.^10^ In total, stool samples from 1117 participants covering an age range from 18 to over 80 years old, were collected at the second visit. The LLD study was approved by the ethics committee of the University Medical Centre Groningen (METc number: 2017/152). All participants signed an informed consent before enrolment.

### Data collection

For GenR and RS, stool samples were collected at home by participants or caregivers and sent to the research center by regular mail with defecation date and information on antibiotics and probiotics use recorded on a questionnaire. Following, bacterial DNA was extracted, 16S rRNA V3–V4 sequenced, and taxonomic classification was reconstructed using the SILVA 16S database release 138.1.^11^ Information for technical covariates such DNA isolation batch, sequencing batch, and TimeInMail (the time length between defecation and sample arrival to the research center) was registered. For LLD, stool samples were collected by students at the University Medical Center Groningen. The 16S rRNA V4 gene region was sequenced and the SILVA 16S database release 128 was used for taxonomic classification.^11^ For all three cohorts, a 10% prevalence cut-off was applied to filter out amplicon sequence variants (ASVs), reducing data sparsity and computation complexity. Detailed information on the collection and bioinformatic processing of the samples per cohort can be found in the Supplementary eMethods.

Information on metabolic traits including anthropometric measurements (i.e., height, body weight, BMI, waist-to-hip ratio, body fat percent, obesity status), blood biochemistry parameters (glucose, insulin, insulin resistance, total cholesterol (TC), high-density lipoprotein-cholesterol (HDL_c), and triglycerides, C-reactive protein (CRP)), systolic and diastolic blood pressure (SBP and DBP), T2D and hypertension (for RS and LLD), was available at time of stool collection. Data on ASCVD (6.5 years follow-up) was available only on RS. Incident ASCVD was defined as a composite outcome comprising fatal or nonfatal myocardial infarction, revascularization (including percutaneous coronary intervention and coronary artery bypass grafting), or stroke. In addition, information on covariates including migration background (for GenR), education (or maternal education for GenR), physical activity, smoking and alcohol intake (for RS and LLD), diet quality score, energy intake, and medication use (i.e., proton pump inhibitors (PPIs) and lipid-lowering agents (for RS and LLD)) was retrieved. Detailed information on the different definitions, measures, assessment, and standardization processes for metabolic traits and lifestyle factors can be found in the Supplementary eMethods. An overview of metabolic phenotypes and covariates available per cohort can be found in Supplementary Table S1.

### Participants

In GenR, stool samples from 1488 participants (mean age 9.8 years, range: 8.8–12.1 years) were successfully sequenced and endured quality control criteria. Whereas 1420 samples from RS participants (mean age 62.7 years, range: 52.4–91.2 years) and 1117 samples from LLD participants (mean age 45.0 years, range: 18–81 years) were successfully processed. Sample inclusion and exclusion criteria can be found in the Supplementary Material eMethods.

### Data analysis

**Fig. 1:**
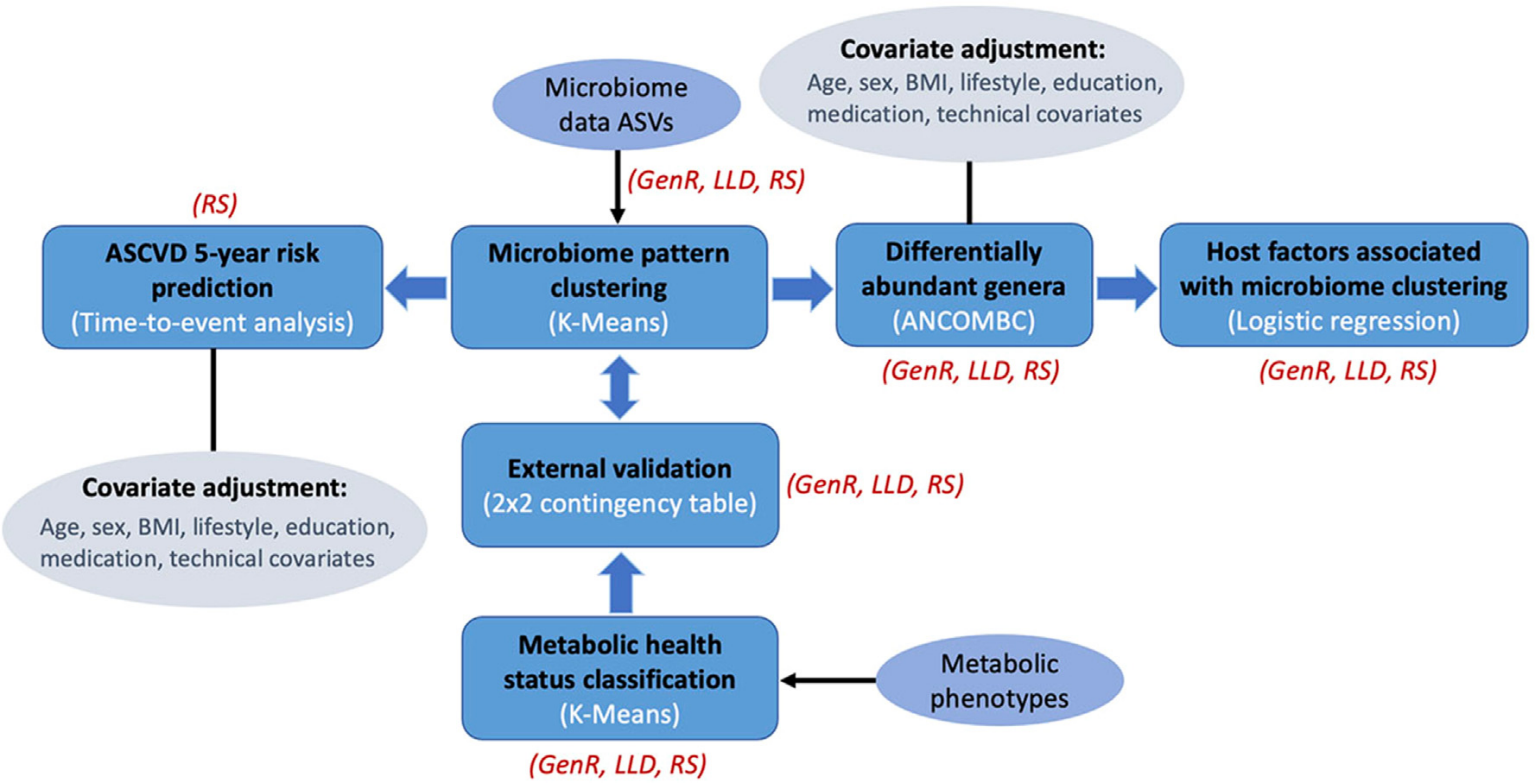
**A schematic figure of analyses performed in this study.** Each frame shows the performed analysis (in black) and correspondent analysis approach (in white); cohorts that were used for performing each of the analyses are also shown (in red) besides the analysis. Microbiome-pattern clustering was performed at the ASV level, while all other association analyses were performed at the genus level.

For this study, a cross-sectional design was used to assess the association between microbiome clustering and metabolic health. Only when investigating a potential association of microbiome clusters and incident ASCVD, in RS, data on cardiovascular events from the stool collection date until January 1st, 2020 was analyzed. Multiple imputation of missing data on study covariates was carried out in RS and GenR. All statistical analyses were carried out in R version 4.4.1. A schematic overview of all analyses performed in this study is shown in Fig. 1.

#### Association of microbiome clusters and metabolic health

Microbiome clustering was performed in each cohort independently with the K-Means algorithm at the ASV level, based on Aitchison's distances across samples. Different cluster quality indexes and random seeds were applied to assess the stability of the assignments (Supplementary eMethods, Supplementary Table S2). Microbiome clustering visualization was carried out by anchoring the data to the two first axes of variation obtained by principal component analysis (PCA). Initial descriptive analyses of demographic and metabolic variables across clusters were performed using the χ2 test and Wilcoxon or Student t-test (for categorical and continuous variables, respectively). Clustering-driving genera were identified after taxonomic classification at the genus level, using ANCOM-BC,^12^ a compositional-aware package with bias correction and adjusting for age, sex, BMI, (maternal) education level, lifestyle factors, medication use (for RS and LLD), and technical covariates (for RS and GenR). Holm-Bonferroni multiple-testing correction was applied based on the number of tested taxa. Host factors associated with the microbiome cluster assignment were identified with multivariable logistic regression models. Briefly, we started with a saturated model where all metabolic phenotypes and possible confounders per cohort were included (see previous section). We then used backward selection for model building using the Akaike information criterion (AIC).

In addition, to validate the microbiome clustering externally, we generated the ground-truth label of each individual's metabolic health status by performing K-Means on a spectrum of selected metabolic phenotypes including age, BMI, WHR (RS and LLD), body fat percentage (GenR and RS), CRP (GenR), glucose and insulin (GenR and RS) levels, TC, HDL_c, triglycerides and blood pressure measurements. A post-hoc cluster validation was performed comparing clinical outcomes between the two metabolic clusters. The association between the microbiome cluster assignment and the metabolic cluster assignment (i.e., health status) was assessed using a 2 × 2 contingency table.

#### Association of microbiome clusters and incidence of ASCVD events

The ASCVD incidence rate per microbiome cluster was calculated in RS using the Clopper-Pearson exact binomial method,^13^ and compared using the Kaplan–Meier, Log-rank test. Cox proportional-hazards models were used to estimate the hazard ratios (HR, 95% CI) adjusting for age, sex, BMI, smoking, alcohol intake, education level, lipids-lowering medication use, and technical covariates. We also calculated the ASCVD 5-year risk for each participant with a re-calibrated pooled risk equation.^14^ Differences in risk were evaluated using a Student t-test.

### Role of the funding source

The funders of the study had no role in study design, data collection, data analysis, interpretation, writing of the report.

## Results

The basic characteristics of the GenR, RS, and LLD participants taking part in this study are shown in Table 1. Overall, microbiome data from GenR participants comprised 80 genera and 330 ASVs, while that from RS comprised 97 genera and 367 ASVs, and the one from LLD 104 genera and 304 ASVs. However, comparing the taxonomic assignment across cohorts revealed that only 74 genera were present both in the GenR and RS cohorts, 68 in the RS and LLD cohorts and 54 in the GenR and LLD.

**Table 1:** Basic characteristics of participants from the Rotterdam Study, the Generation R Study and the Lifelines-DEEP Study.

	Generation R Study (n = 1488)	Rotterdam Study (n = 1265)	Lifelines-DEEP Study (n = 1117)
Age (mean ± sd) (years)	9.83 ± 0.31	62.68 ± 5.67	45.00 ± 13.70
Sex (male, %)	732 (49.20%)	531 (41.70%)	460 (41.20%)
BMI (mean ± sd)	17.32 ± 2.50	27.27 ± 4.31	25.20 ± 4.10
Smoking rate	NA	158 (12.50%)	218 (19.70%)
Alcohol, median (IQR) (g/day)	NA	6.43 (1.61–8.57)	6.30 (1.70–12.10)
Energy intake, median (IQR) (kcal/day)	1469.45 (1244.97–1735.75)	2255.33 (1856.10–2724.82)	1938.10 (1606.50–2355.70)
Diet quality score (mean ± sd)	4.52 ± 1.25	7.07 ± 1.86	NA
Physical activity, median (IQR) (MET score or hours/week)^a^	10.12 (7.12–13.88)	49.77 (22.20–85.30)	6.00 (2.80–12.00)
(Maternal) Education level^b^			
Low	52 (3.50%)	508 (40.20%)	15 (1.30%)
Medium	510 (34.30%)	356 (28.10%)	566 (50.70%)
High	926 (62.20%)	401 (31.70%)	497 (44.50%)
PPIs use (%)	NA	243 (19.20%)	92 (8.20%)
Lipid lowering medication use (%)	NA	336 (26.60%)	51 (4.60%)
Alpha diversity (Shannon index) (mean ± sd)	3.97 ± 0.42	3.98 ± 0.44	3.74 ± 0.44

“NA”: Data not available.

a Physical activity (hours/week) in the Rotterdam Study is weighted by Metabolic Equivalent of Task (MET) score based on different levels of intensity; in GenR it refers to time spent per week (hours); in Lifelines-DEEP it refers to the time of moderate and rigorous physical activity per week.

b Educational level for the Rotterdam Study and Lifelines-DEEP, and maternal educational level for the Generation R Study.

### Microbiome-derived clustering

**Fig. 2:**
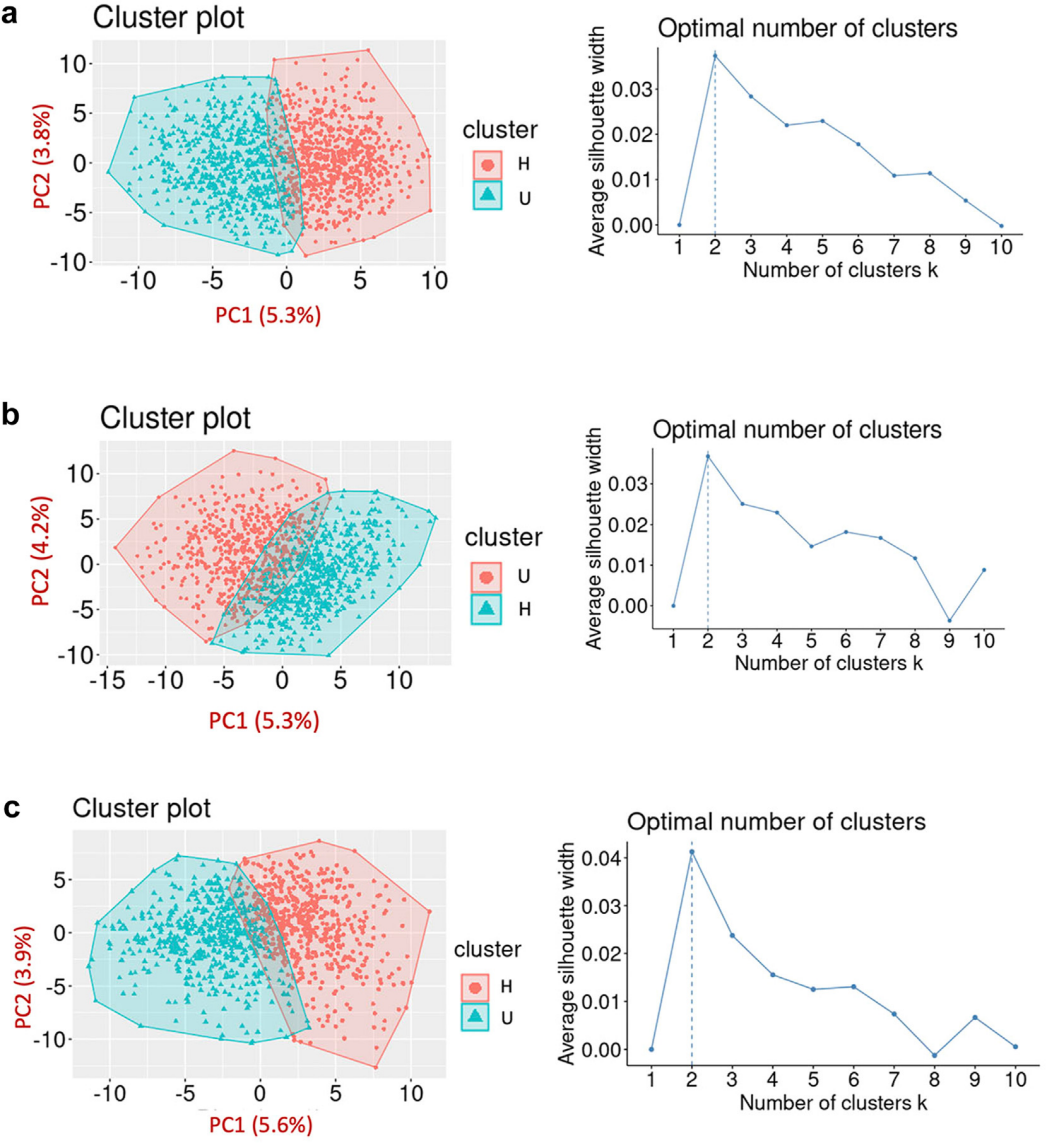
**Visualization of two clusters in the background of the ASV's principal component analysis within each cohort (a) the Generation R Study, (b) the Rotterdam Study, and (c) the Lifelines-DEEP Study.** Visualization of the first two principal components derived from the corresponding ASV tables. Coloring on the figures corresponds to the cluster assignment by the K-means algorithm. For each cohort, the results of the average silhouette width algorithm to select the optimal number of clusters are shown in the right panels.

Based on the microbiome ASV information, the K-means algorithm robustly identified two distinct clusters as the best partition to describe the microbiome data of each cohort (Fig. 2). Metabolic phenotypes, as well as sociodemographic and lifestyle factors of the participants assigned to either cluster differed substantially (Table 2, Table 3). Hence, the cluster in which clinical outcomes and lifestyle behaviors were generally healthier, considering the different age groups, was labeled cluster H, a healthier group. Correspondingly, the other cluster was labeled cluster U, an unhealthier group.

**Table 2:** Comparison of metabolic phenotypes between individuals assigned to different microbiome clusters.

	Generation R Study (n = 1488)			Rotterdam Study (n = 1265)			Lifelines-DEEP Study (N = 1117)		
	Cluster H (n = 806)	Cluster U (n = 682)	p value	Cluster H (n = 578)	Cluster U (n = 687)	p value	Cluster H (n = 536)	Cluster U (n = 581)	p value
Age (years)	9.82 ± 0.33	9.83 ± 0.30	0.04∗	62.38 ± 5.47	62.93 ± 5.83	0.10	47.40 ± 13.50	42.70 ± 13.50	<0.001∗∗∗
Sex (male,%)	420 (52.10%)	336 (49.30%)	0.30	243 (42.20%)	285 (41.40%)	0.89	217 (40.50%)	243 (41.80%)	0.67
Ethnicity									
European	651 (80.80%)	523 (76.70%)	0.02∗	NA	NA	NA	NA	NA	NA
Asian	36 (4.50%)	56 (8.20%)		NA	NA		NA	NA	
African	112 (13.90%)	95 (13.90%)		NA	NA		NA	NA	
Other	7 (0.90%)	8 (1.20%)		NA	NA		NA	NA	
BMI	17.10 ± 2.40	17.57 ± 2.60	<0.001∗∗∗	26.69 ± 4.05	27.75 ± 4.46	<0.001∗∗∗	24.70 ± 3.60	25.60 ± 4.50	<0.001∗∗∗
Overweight prevalence	12.90% (104)	18.90% (129)	<0.01∗∗	44.50% (257)	46.00% (316)	0.62	39.90% (214)	50.90% (296)	<0.001∗∗∗
Obesity prevalence	1.90% (15)	2.80% (19)	0.30	18.70% (108)	27.10% (186)	<0.001∗∗∗	7.80% (42)	15.10% (88)	<0.001∗∗∗
Body fat (%)	28.74 ± 6.46	30.30 ± 6.78	<0.001∗∗∗	34.17 ± 7.22	36.29 ± 7.22	<0.001∗∗∗	NA	NA	NA
Waist-to-hip ratio	NA	NA	NA	0.88 ± 0.10	0.90 ± 0.09	<0.001∗∗∗	0.91 ± 0.080	0.92 ± 0.080	0.44
Glucose (mmol/L)	5.24 ± 0.97	5.31 ± 0.95	0.12	5.57 ± 0.87	5.81 ± 1.15	<0.001∗∗∗	5.00 ± 0.70	4.90 ± 0.70	0.82
Insulin (log-) (pmol/L)	5.10 ± 0.75	5.16 ± 0.72	0.14	1.83 ± 0.24	1.89 ± 0.26	<0.001∗∗∗	NA	NA	NA
Insulin resistance	NA	NA	NA	3.00 ± 3.10	3.78 ± 4.05	<0.001∗∗∗	2.00 ± 1.10	2.60 ± 1.80	<0.001∗∗∗
T2D prevalence	NA	NA	NA	8.40% (49)	15.30% (105)	<0.001∗∗∗	2.10% (11)	1.80% (10)	0.83
Cholesterol (mmol/L)	4.26 ± 0.60	4.32 ± 0.66	0.10	5.65 ± 1.09	5.55 ± 1.10	0.14	5.08 ± 1.00	4.99 ± 1.01	0.16
Triglycerides (log_10_-) (mmol/L)	−0.06 ± 0.45	−0.00014 ± 0.46	<0.001∗∗∗	0.085 ± 0.18	0.14 ± 0.20	<0.001∗∗	−0.048 ± 0.21	0.021 ± 0.23	<0.001∗∗∗
HDL_c (mmol/L)	1.50 ± 0.34	1.50 ± 0.33	0.95	1.54 ± 0.45	1.49 ± 0.46	0.02∗	1.60 ± 0.42	1.50 ± 0.40	<0.001∗∗∗
Mean SBP (mmHg)	102.18 ± 7.77	103.33 ± 7.77	<0.01∗∗	132.82 ± 18.80	135.40 ± 18.01	<0.01∗∗	118.20 ± 13.20	119.60 ± 13.50	0.13
Mean DBP (mmHg)	58.07 ± 6.59	58.57 ± 6.31	0.13	81.43 ± 11.28	82.09 ± 10.50	0.23	70.60 ± 9.00	71.10 ± 9.30	0.32
Hypertension prevalence	NA	NA	NA	54.80% (318)	61.50% (422)	0.02∗	18.00% (96)	21.10% (123)	0.22
CRP									
<1 mg/L	83.40% (672)	79.80% (544)	0.13	NA	NA	NA	28.90% (138)	22.80% (118)	<0.001∗∗∗
1–3 mg/L	12.00% (97)	6.60% (45)					53.00% (253)	49.10% (254)	
>3 mg/L	4.60% (37)	13.60% (93)					18.00% (86)	28.00% (145)	

“∗”: p < 0.05; “∗∗”: p < 0.01; “∗∗∗”: p < 0.001; “NA”: Data not available.

Results are reported for the three participant cohorts, the Rotterdam Study, the Generation R Study and the Lifelines-DEEP Study.

**Table 3:** Comparison of lifestyle, socioeconomic factors, and medication use between individuals assigned to different microbiome clusters.

	Generation R Study (n = 1488)			Rotterdam Study (n = 1265)			Lifelines-DEEP Study (n = 1117)		
	Cluster H (n = 806)	Cluster U (n = 682)	p value	Cluster H (n = 578)	Cluster U (n = 687)	p value	Cluster H (n = 536)	Cluster U (n = 581)	p value
Energy intake (kcal/day)^a^	1518.37 ± 385.11	1500.39 ± 391.48	0.33	2347.22 ± 666.19	2300.02 ± 687.50	0.25	3.30 ± 0.10	3.30 ± 0.10	0.75
Diet quality score	4.57 ± 1.23	4.47 ± 1.27	0.11	7.27 ± 1.90	6.91 ± 1.82	<0.001∗∗∗	NA	NA	NA
Physical activity (MET score or hours/week)^b^	11.26 ± 5.94	11.01 ± 5.70	0.40	63.00 ± 51.00	61.60 ± 60.80	0.050	9.70 ± 10.40	9.10 ± 10.10	0.34
Smoking rate	NA	NA	NA	10.00% (57)	14.70% (101)	0.01∗	19.30% (103)	29.80% (173)	0.0040∗∗
Alcohol (g/day)^c^	NA	NA	NA	7.83 ± 7.87	7.88 ± 8.74	0.21	1.80 ± 1.10	1.80 ± 1.00	0.69
Education^d^									
Low	20 (2.50%)	32 (4.70%)	<0.001∗∗∗	201 (34.70%)	307 (44.60%)	<0.001∗∗∗	8 (1.60%)	7 (1.20%)	0.011∗
Medium	247 (30.70%)	263 (38.60%)		165 (28.60%)	191 (27.90%)		245 (47.80%)	321 (56.80%)	
High	539 (66.90%)	387 (56.70%)		212 (36.70%)	189 (27.50%)		260 (50.70%)	237 (41.90%)	
PPIs use rate	NA	NA	NA	15.50% (90)	22.20% (153)	<0.01∗∗	6.50% (35)	9.80% (57)	0.050
Lipids lowering drugs (statins) use rate	NA	NA	NA	21.60% (125)	30.70% (211)	<0.001∗∗∗	3.20% (17)	5.90% (34)	0.044

“∗”: p < 0.05; “∗∗”: p < 0.01; “∗∗∗”: p < 0.001; “NA”: Data not available.

Results are reported for the three participant cohorts, the Rotterdam Study, the Generation R Study and the Lifelines-DEEP Study.

a Log-scale transformation of energy intake for Lifelines-DEEP Study.

b Physical activity (hours/week) in RS is weighted by Metabolic Equivalent of Task (MET) score based on different levels of intensity; in GenR it refers to time spent per week (hours); in Lifelines-DEEP it refers to the time of moderate and rigorous physical activity per week.

c Alcohol intake is transformed by log (g/day + 1) in the Lifelines-DEEP Study.

d For GenR, it refers to maternal education level; for RS and LLD, it refers to education level.

In the pediatric cohort, we observed that participants in cluster U presented higher BMI, body fat percentage, triglycerides and CRP levels, and SBP (all p < 0.05; Table 2). Average maternal education and migration background also differed across participants in both clusters. In RS, participants assigned to cluster U had significantly (p < 0.05) higher BMI, waist-to-hip ratio, body fat percentage, HDL_c, triglycerides, glucose and insulin levels, insulin resistance, SBP, and, were more often diagnosed with T2D and hypertension, lower diet quality scores, higher smoking rate, relatively lower education level, and were more often users of proton pump inhibitors (PPIs) and lipid-lowering medication (p < 0.05). We observed similar patterns in the validation cohort LLD (Table 2, Table 3) While being younger as compared to participants in cluster H, LLD participants in cluster U had significantly (p < 0.05) higher BMI, triglycerides, CRP and insulin levels and insulin resistance, while presenting lower HDL_c. Besides, they were more often smokers and users of lipid-lowering medication as compared to individuals in cluster H (p < 0.05; Table 3).

The microbiome diversity (Shannon index) was also significantly lower (p < 0.05) in cluster U participants as compared to that of participants assigned to cluster H in all three cohorts (3.97 ± 0.41 vs 3.98 ± 0.43 in GenR; 3.80 ± 0.40 vs 4.20 ± 0.40 in RS, and 3.60 ± 0.50 vs 3.90 ± 0.30 in LLD).

### Genera-driving cluster assignment

**Fig. 3:**
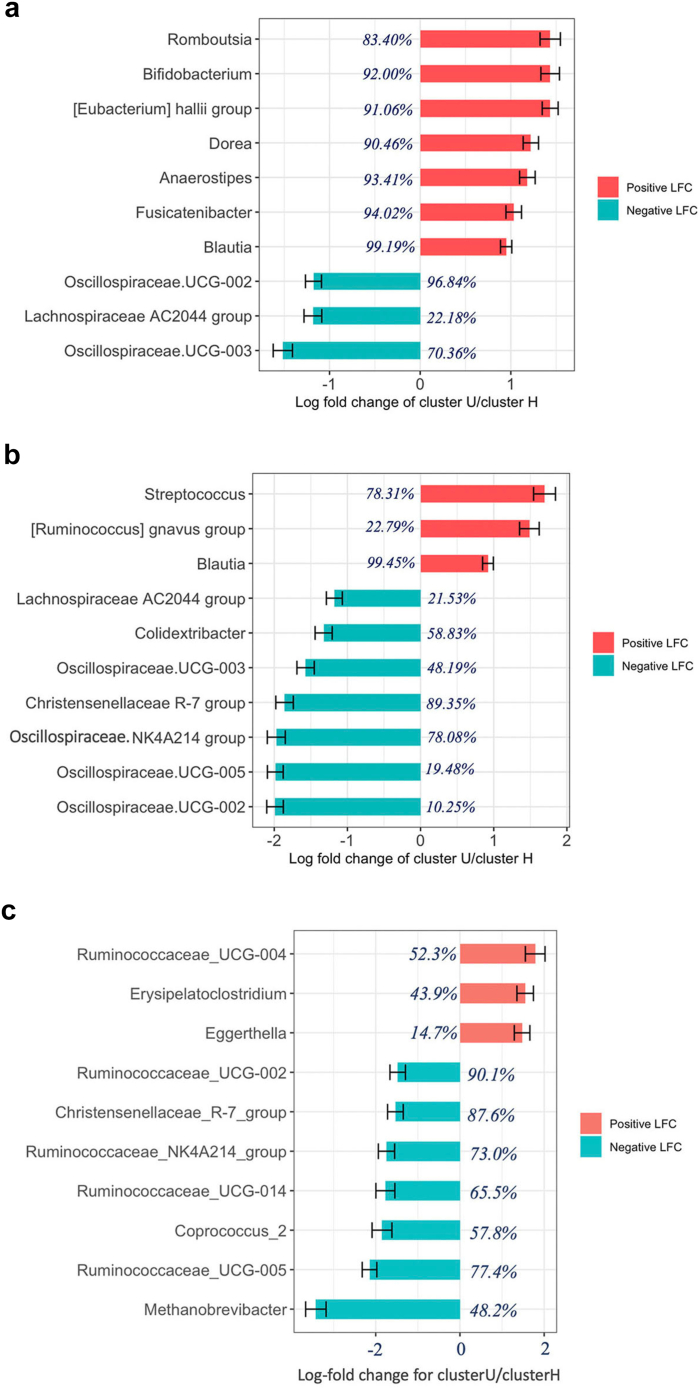
**Top genera contributing to cluster assignment.** Top 10 genera contributing to cluster assignment within (a) the Rotterdam Study, (b) the Generation R Study, and (c) the Lifelines-DEEP Study. The prevalence of each genus is reported as a percentage on the corresponding study population. Bars represent the log fold change (LFC) of cluster U with respect to cluster H. Red bars represent genera with a higher abundance in cluster U, whereas cyan bars represent genera with a lower abundance in cluster U.

**Fig. 4:**
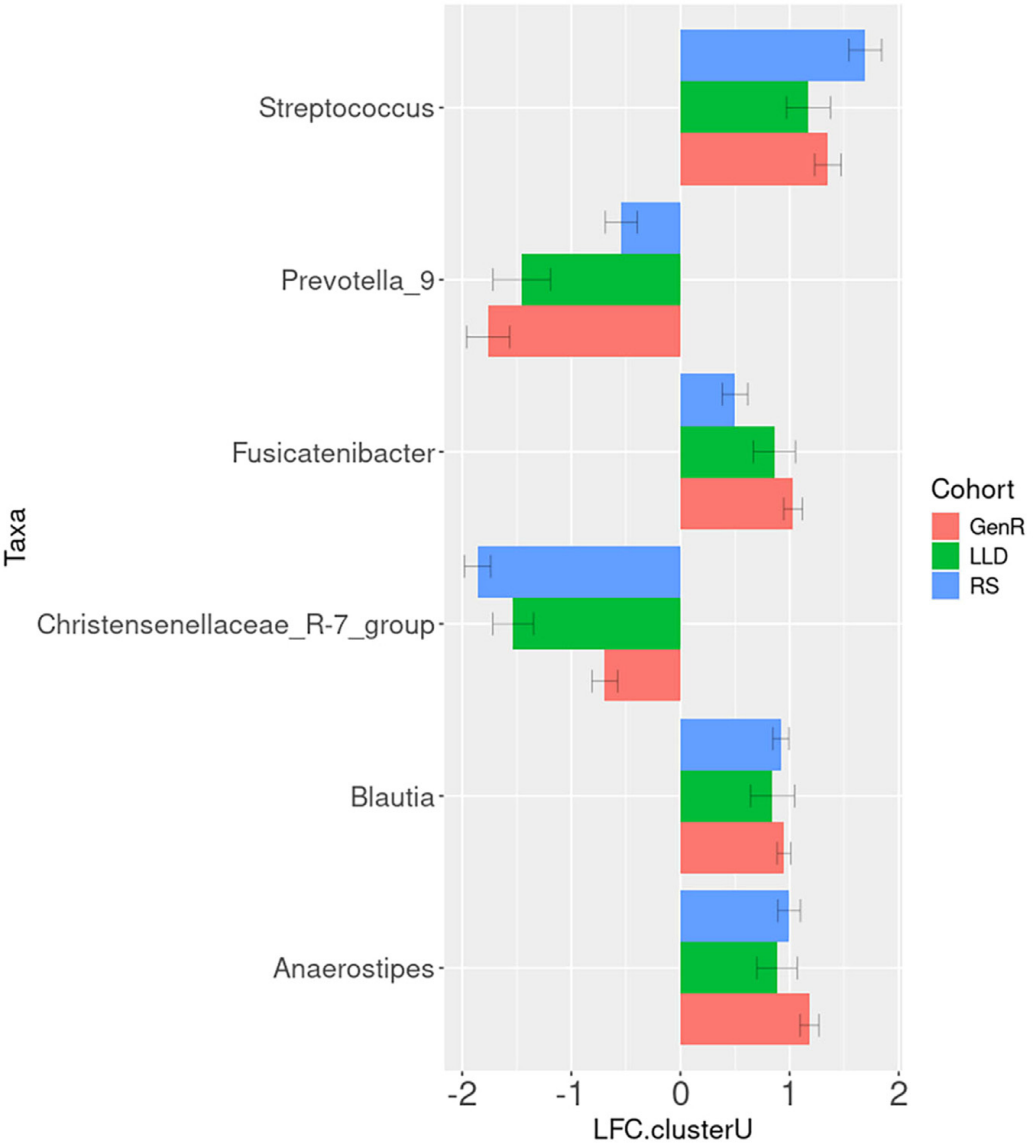
**Overlapped differentially abundant genera across the three cohorts.** Bars represent log-fold change in genera abundance for cluster U (cluster H used as reference). Red bars refer to the Generation R Study, green bars refer to the Lifelines-DEEP Study, and blue bars refer to the Rotterdam Study. “LFC.clusterU”, Log-fold change for cluster U compared to cluster H.

After correcting for possible confounders, we identified (FDR <0.05) 56 (80%) differentially abundant (DA) genera associated with cluster assignment in GenR, 53 (54.6%) in RS, and 47 (45.2%) in LLD (Fig. 3; Supplementary Tables S4–S6). From these significantly associated genera, only six overlapped across the three cohorts, all presenting directional consistency i.e., *Streptococcus*, *Prevotella_9*, *Fusicatenibacter*, *Christensenellaceae_R-7_group*, *Blautia*, and *Anaerostipes* (Fig. 4).

### Host factors associated with microbiome clustering

**Fig. 5:**
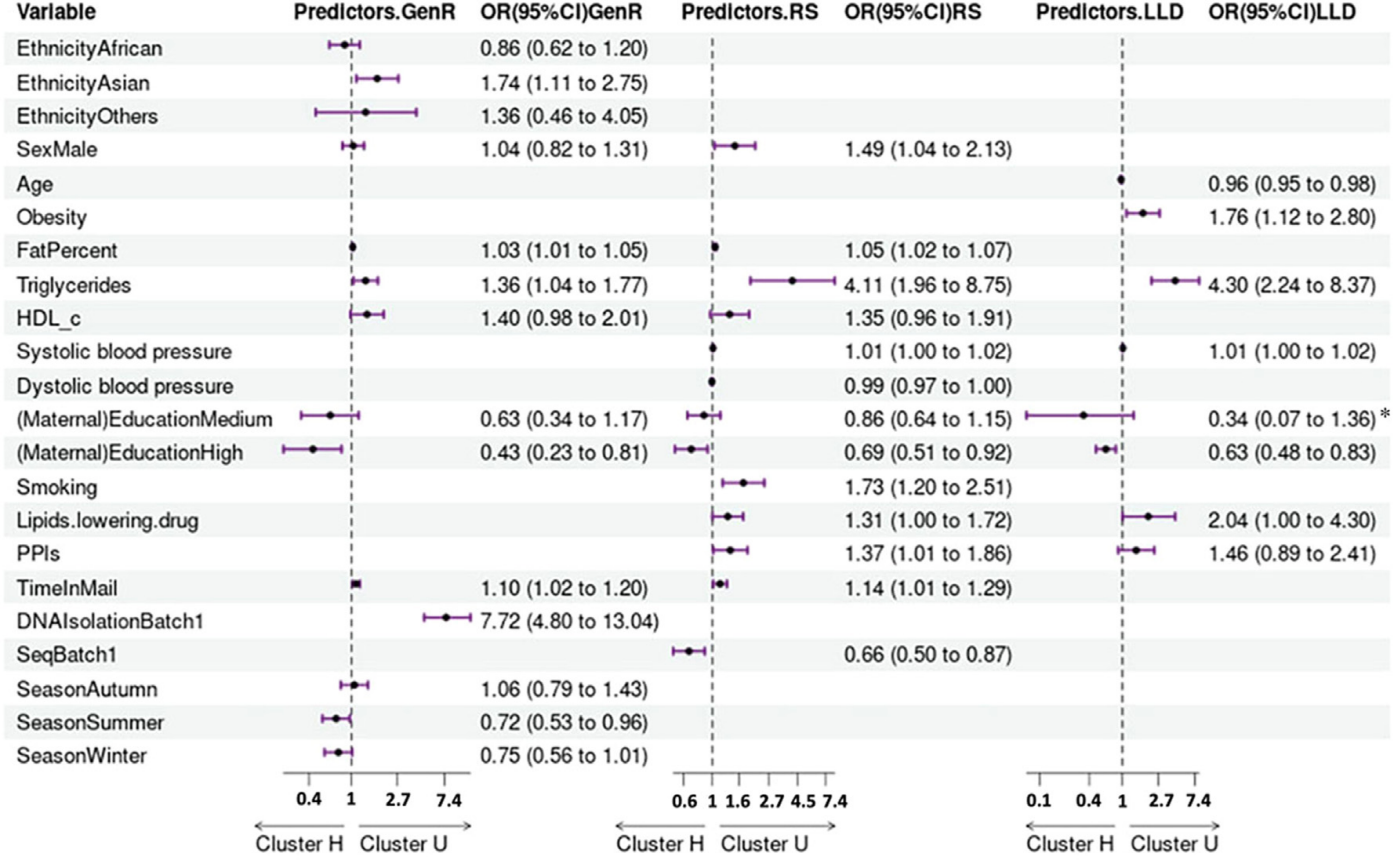
**Forest plot visualizing independent risk factors associated with an unhealthy microbiome cluster derived from logistic regression model for each cohort.** The black diamond in the middle of the purple line represents the effect size of the association, and the purple line represents the 95% confidence interval. Within each column, no data indicates the corresponding variable is either missing in the final model or not significant. “∗” the reported OR (95%) CI corresponds to low education level, as medium level was used as reference for LLD. GenR, Generation R Study; RS, Rotterdam Study, and LLD, Lifelines-DEEP Study.

In GenR, factors positively associated with an unhealthy microbiome cluster assignment included, ethnicity (OR_Asian/European_ = 1.74, 95% CI [1.11, 2.76]) and body fat percentage (OR = 1.03, 95% CI [1.01, 1.05]), whereas maternal education (OR_high/low_ = 0.43, 95% CI [0.23, 0.81]) was inversely associated with an unhealthy microbiome cluster assignment (Supplementary Table S7, Fig. 5). Other technical variables associated with U-cluster assignment were sampling season (OR_summer/spring_ = 0.72, 95% CI [0.53, 0.96]), time in mail (OR = 1.10, 95% CI [1.02, 1.20]) and DNA isolation batch (OR = 7.72, 95% CI [4.80, 13.04]). In RS, being a male (OR = 1.49, 95% CI [1.04, 2.13]), smoking (OR = 1.73, 95% CI [1.20, 2.51]), being a PPIs user (OR = 1.37, 95% CI [1.01, 1.86]), having a high body fat percentage (OR = 1.05, 95% CI [1.02, 1.07]) or high triglycerides (OR = 4.11, 95% CI [1.96, 8.75]) associated with an unhealthy microbiome cluster assignment, whereas education level (OR_high/low_ = 0.69, 95% CI [0.51, 0.92]) was protective of U-cluster assignment (Supplementary Table S8, Fig. 5). Technical covariates such as time in mail (OR = 1.14, 95% CI [1.01, 1.29]) and sequencing batch (OR = 0.66, 95% CI [0.50, 0.87]) also impacted U-cluster assignment. In the validation cohort (a subset of LLD with complete data, n = 972), being obese (OR = 1.76, 95% CI [1.12, 2.80]) and having high triglyceride levels (OR = 4.30, 95% CI [2.24, 8.37]) were associated with U-cluster assignment. Whereas, younger age (OR = 0.96, 95% CI [0.95, 0.98]), and education level (OR_high/medium_ = 0.63, 95% CI [0.48, 0.83]) were negatively associated with an unhealthy microbiome assignment (Supplementary Table S9, Fig. 5).

### Derived metabolic health status

We applied K-means clustering once again, but this time to a collection of anthropometric and metabolic phenotypes, and identified two distinctive metabolic clusters within each study population (Supplementary Figure S4). As expected, the two metabolic clusters showed very distinctive patterns in a broad spectrum of metabolic phenotypes, including age, sex, BMI, body fat percentage, waist-to-hip ratio, CRP, lipid, glucose and insulin levels, blood pressure measurements and prevalence of T2D or hypertension (Supplementary Table S12). Similar to the microbiome-based clustering, we renamed the two metabolic-derived clusters as “cluster-H” (healthy) or “cluster-U” (unhealthy) based on their relation with metabolic status.

#### Association of microbiome clusters and metabolic health status

We evaluated the likelihood of an individual to have an unhealthy metabolic status given the microbiome cluster he belonged to. Children assigned to cluster-U had higher odds of being metabolically unhealthy (OR = 1.33, 95% CI [1.07, 1.65]). For RS, the odds for individuals assigned to the microbiome cluster-U to be metabolically unhealthy were 1.61 times higher than the odds for those assigned to microbiome cluster-H (OR = 1.61, 95% CI [1.29, 2.01]). The odds of being metabolically unhealthy were also higher for U-cluster participants from LLD (OR = 1.15, 95% CI [0.88, 1.50]) (Supplementary Table S10).

### Gut microbiome cluster assignment association with ASCVD risk

**Fig. 6:**
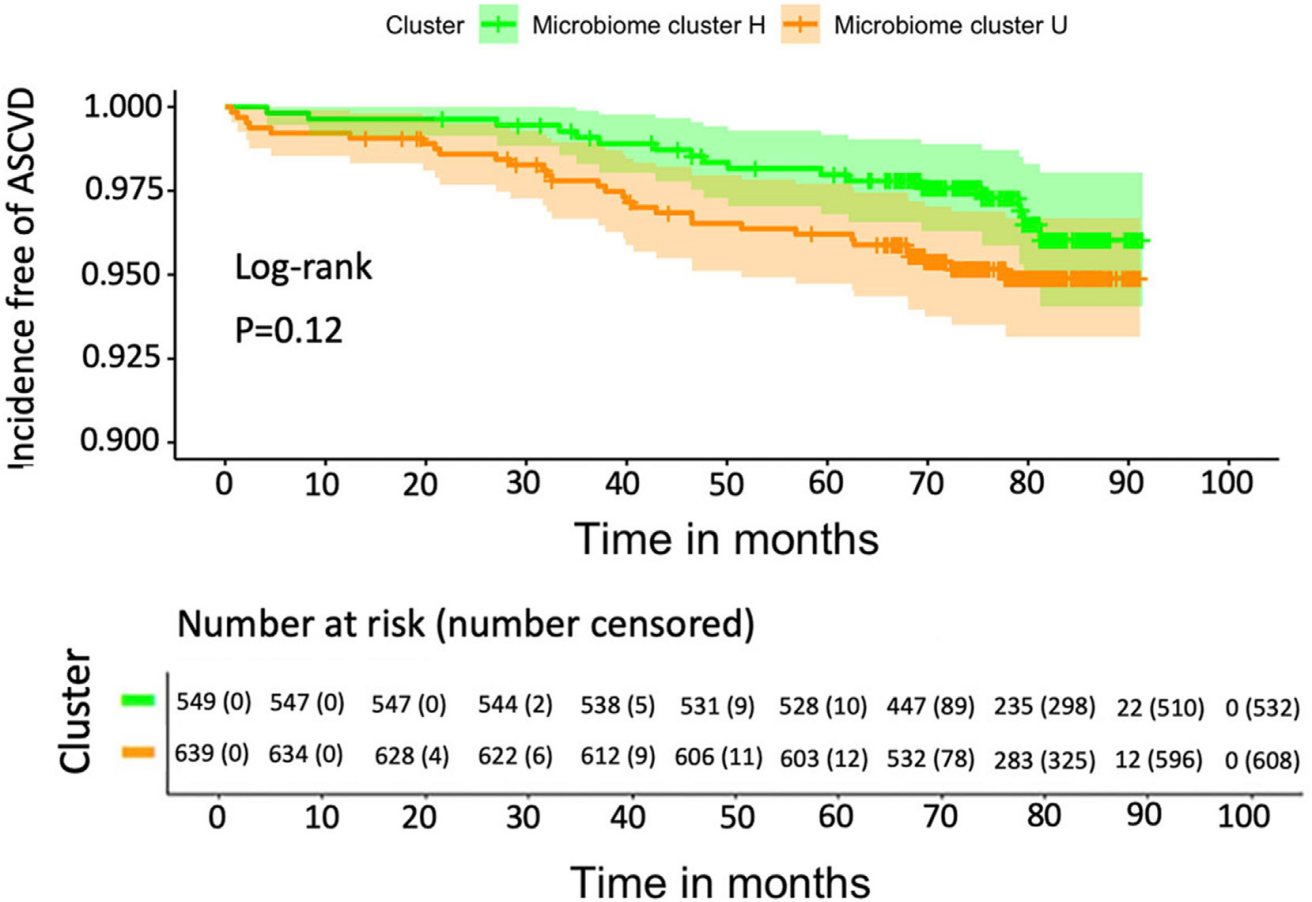
**Kaplan Meier curves of incidence-free atherosclerotic cardiovascular disease (ASCVD) by microbiome cluster in the Rotterdam study.** The x-axis denotes follow-up time in months, and the y-axis denotes the proportion of individuals who did not get incident ASCVD at each of the follow-up time points. The number of individuals who were at risk of ASCVD at each time point is presented in the lower panel. The green curve represents individuals from microbiome cluster H, and the yellow one represents individuals from microbiome cluster U.

The ASCVD incidence rate in RS (n = 1188) was 6.37 (95% CI [4.70–8.45]) per 1000 person-years. Partitioning the data based on the microbiome clusters resulted in a higher incident rate for U-cluster participants, estimated as 7.71 (95% CI [5.24–10.94]) per 1000 person-years than for those in cluster H (4.84 (95% CI [2.82–7.75]) per 1000 person-years). During the follow-up, the probability of incident ASCVD events was higher in microbiome cluster U than in cluster H, yet, this difference was not statistically significant (log-rank test p > 0.05) (Fig. 6). During a median follow-up time of 6.5 (interquartile range: 6.0–6.9) years, the adjusted hazard ratio for ASCVD events for those in microbiome cluster U was 1.52 (95% CI [0.83, 2.80]) times higher than for those in cluster-H (Supplementary Table S11). Whereas the mean 5-year ASCVD risk (i.e., probability of developing ASCVD) was 0.054 (SD: 0.060) for the whole population and differed (p < 0.001) across microbiome clusters (0.059 (SD: 0.071) for cluster U and 0.047 (SD: 0.042) for cluster H).

## Discussion

Following a life-course approach, our study evaluated the relationship between human gut microbiome profiles and metabolic health in the general population. We analyzed data from 2753 individuals from two different life stages, pre-adolescence (the Generation R Study) and older adults (the Rotterdam Study). The microbiome data of these individuals was processed simultaneously at the laboratory using similar protocols. Despite the innate differences of these cohorts, we were able to identify two distinct microbiome clusters in each cohort. Our results revealed these microbiome clusters were intrinsically associated with health status by analyzing metabolic phenotypes. Similar results were obtained in an external cohort (LLD) of 1117 adults from Groningen, The Netherlands. Yet, the association between microbiome patterns and metabolic health was not statistically significant in LLD, probably due to its smaller sample size. We identified DA genera that contributed to cluster assignment throughout the life- course. In the discovery cohorts from 32 DA genera present in adolescents and old adults, 93% were consistent in the direction of the effect. This high overlap highlights the existence of microbial taxa whose link with overall metabolic status seems independent of the dramatic impact of aging, in other words, bacteria present already in early puberty might be definitive in shaping metabolic health later in life. Remarkably, the odds of displaying an unhealthy metabolic profile were between 33 and 66% higher in adolescents and old adults presenting an unhealthy microbiome profile as compared to those who did not. Particularly, participants presenting an unhealthy microbiome profile also presented a higher 5-year ASCVD risk. It is important to note that determinants of health vary across the life-course. For instance, children at peripubertal age are not expected to smoke or consume alcohol nor are they normally prescribed PPIs or lipid-lowering agents.

Our results align with the contention that a particular gut microbiome profile can reflect metabolic health status regardless of the age of assessment. The human gut microbiome changes significantly over the lifespan, and microbiome-mediated effects on health are influenced by age-specific differences.^3^ In our study, we observed a stronger association between cluster assignment and metabolic health in the old adults as compared to the adolescents. One plausible explanation is that children and adolescents still have an immature gut microbiome.^8^ Yet, changes in lifestyle (e.g., smoking, alcohol use) across life stages, or the varying exposure to sex-related hormones could contribute to these differences. Notwithstanding differences in measure assessments or instruments used to evaluate clinical outcomes, we observed a high correlation between body fat percentage and serum triglycerides with microbiome cluster assignment in the three cohorts. Besides metabolic phenotypes, socioeconomic status (i.e., education or maternal education level) also impacted microbiome cluster assignment in line with previous studies.^15^^,^^16^ The higher educational level usually translates into healthier lifestyles, better social support, and better access to health services, all of which would contribute to an overall higher healthy status. Other lifestyle factors, previously associated with microbiome dysbiosis such as smoking^17^ and use of antiacids^18^ were higher in individuals assigned to the unhealthy microbiome cluster. In addition, ethnicity, assessed only in Generation R, also showed an association with microbiome cluster assignment. Previously, in the same cohort, migration background disparities in screen-watching time,^19^ levels of physical activity, and diet quality^20^ which could disrupt the gut microbiome, have been described. Even if the role of host genetics on the microbiome composition has been deemed to be relatively small,^6^ a diverse genetic background could also contribute to the observed differences.

Differentially abundant genera across the microbiome clusters have been reported as associated with metabolic phenotypes. *Christensenellaceae R-7 group*, a metabolically beneficial taxon,^21^ was elevated in the healthy microbiome cluster across all investigated cohorts. *Prevotella-9* seemingly representing a strain of *Prevotella copri*,^22^ a genera with contradictory health effects,^23^ was also more abundant in the healthier microbiome cluster. Whereas *Streptococcus, Fusicatenibacter, Blautia* and *Anaerostipes* were increased in individuals assigned to the unhealthy microbiome cluster. *Streptococcus* is associated with inflammation markers and coronary atherosclerosis.^24^ High relative abundance of *Fusicatenibacter* has been correlated with unhealthy eating behavior^25^ and insomnia disorders.^26^ Scientific reports have presented conflicting evidence regarding the impact of *Blautia* on human health, with decreased abundance associated with visceral fat accumulation, diabetes and Crohn's disease, but also increased abundance reported in certain cancers.^27^ *Anaerostipes* have a butyrogenic potential but may synthesize fatty acids and metabolize anticancer agents.^28^ Overall, the evidence presented here constitutes only a selection of the numerous microbiome studies in which these genera have shown associations with human health. Yet, it is important to note that 16S rRNA sequencing analysis provides low taxonomical resolution and falls short in pinpointing exact species or strains, which can present diverse metabolic potential.

It is important to mention that the overlap in mapped taxonomies across cohorts was not high, limiting the power to identify a unique health microbiome signature. We speculate there are a few reasons for this. While the Generation R Study is a pediatric cohort representing the diverse migration backgrounds present in the city of Rotterdam, the Rotterdam Study focuses on old adults, mainly of Dutch background, inhabitants of the district of Ommord. In contrast, Lifelines includes only participants able to read Dutch and is conducted in the three northern provinces of The Netherlands, where, historically, migration has been lower.^29^ These differences might result in a specific distribution of the microbial communities in one or another cohort. Another explanation for the observed proportions of overlapped taxa would be that the discovery and replication cohorts differed in sequenced 16S variable region and taxonomic reference dataset used, which could result in a different taxonomic classification for the same bacteria.^30^ Furthermore, taxa can have redundant functional potential and thus the gut microbiome of people can be different in terms of composition but still yield similar protein and metabolic profiles.^31^ Above these differences, a healthy microbiota was described by a high taxonomic diversity in all three cohorts, as expected.

To our knowledge, this is the first study exploring a life–course relationship between gut microbiome profiles and metabolic health in the general population. Even if the inclusion of different cohorts strengthens the generalizability and robustness of the findings, our study also has limitations not discussed yet, that need to be acknowledged. We were confined by the use of 16S rRNA sequencing and low statistical power to investigate taxa at a higher resolution or survey the association of microbial genes and pathways with host metabolic health. In the discovery cohorts, dietary data was registered two to five years before stool collection. Therefore, potential changes in dietary behavior over time and seasons could not be accounted for in our study. However, we expect that the adjustment for deposition season in our statistical models partially mitigates the artifacts introduced by this limitation. Moreover, besides the lack of data on cardiovascular events in LLD, given the short follow-up time in RS, the ASCVD 5-year risk was calculated using a formula developed by the ACC/AHA guidelines for a 10-year ASCVD risk calculation.^14^ Lastly, our study lacked a proper representation of infants and elderly individuals, emboding life stages of broad microbiome changes. Therefore, our findings should not be directly generalized to these groups. Also, even if one of the participating cohorts comprised individuals of diverse ethnicities, all three cohorts were conducted in the Netherlands. Pooling participant cohorts across countries, particularly, including racially/ethnically diverse populations, would improve the generalizability of our results and facilitate intra and inter-population comparisons. Future studies are warranted to investigate the mechanisms underlying the gut microbiome's influence on metabolic health, for example, using animal experiments of stool microbiota transplantation, or designing interventional studies.

In conclusion, our study revealed that gut microbiome clusters are linked to different metabolic health statuses across the life-course. Metabolic phenotypes, including body fat percentage and triglycerides, and socioeconomic level strongly predict microbiome cluster assignment. Moreover, our study showed that, in older adults, an unhealthy microbiome pattern could be an early indication of an increased ASCVD risk in future years. As the gut microbiota is more susceptible to modulation by environmental factors, such as diet, medication, lifestyle, and geographical environment, compared to host-intrinsic factors, such as genetics, our study postulates that targeting the microbiome might be an effective intervention to promote a better metabolic health status. More specifically, childhood and adolescence might provide an extra opportunity for the prevention of metabolic disorders across the lifespan.

## Contributors

Study concept and design R.L., F.R., M.K., C.M-G. Acquisition of data and data preparation for analysis A.K., J.A.E.v O, F.A, R.K., L.D, J.F., K.I., M.K. Statistical analyses R.L., A.K., S.Y., A.v H., A.Y. Interpretation of the data R.L., S.Y., A. v. H., G.R., M.K., A.Z., F.R., C. M-G. Drafting of the manuscript R.L., F.R., C.M-G. Administrative or technical support R.K. Funding acquisition: R.K., J.F., V.W.V.J, A.G.U., A.Z. All authors have critically assessed the manuscript and provided feedback. R.L. and C.M-G have directly accessed and verified the underlying data reported in the manuscript and take responsibility for the integrity of the data and the accuracy of the data analysis.

## Data sharing statement

The Lifelines datasets can be obtained by submitting a proposal, all information can be found on its website (https://www.lifelines-biobank.com/researchers/working-with-us/step-1-prepare-and-submit-your-application). In the case of the Generation R Study and Rotterdam Study, the data set cannot be shared in public repositories given the constrains in the participants informed. However, data and analytical scripts can be obtained upon request to the correspondent author.

## Declaration of interests

Liesbeth Duijts declares receiving research funding from the PROTEA-2 study, ENDOMIX project European Union's Horizon 2020 research and innovation program no. 101136566, and the Asthma Control Foundation, and speaker fees from Astra Zeneca, British Thoracic Society, European Respiratory Society, Karolinska Institute, University of Copenhagen, Barcelona Institute for Global Health. All other authors declare no competing interests.

All authors confirm they had full access to all the data in the study and accept responsibility to submit for publication.
