# Supplementary material for: Association between gut microbiome profiles and host metabolic health across the life course: a population-based study
Source: Lancet Reg Health Eur. 2024 Dec 28;50:101195. doi: 10.1016/j.lanepe.2024.101195 (PMC11743806; doi:10.1016/j.lanepe.2024.101195)
Supplement: Supplementary material [file mmc1.pdf]

Supplementary Material

Association between gut microbiome profile and host metabolic health across the life course: a population-based study

Ruolin Li et al.

Supplementary Material: eMethods **Page 3**

Supplementary Table S1 **Page 10**
An overview of metabolic phenotypes and covariates data available per cohort, and variables used for each step analysis.

Supplementary Table S2 **Page 11**
Best cluster number determination for microbiome-ASV based clustering based on three different algorithms

Supplementary Table S3 **Page 12**
Best cluster number determination for metabolic phenotype data-based clustering based on three different algorithms

Supplementary Table S4 **Page 13**
Differential abundant taxa between clusters of Generation R Study identified by ANCOM-BC

Supplementary Table S5 **Page 15**
Differential abundant taxa between clusters of Rotterdam Study identified by ANCOM-BC

Supplementary Table S6 **Page 18**
Differential abundant taxa between clusters of Lifelines-DEEP Study identified by ANCOM-BC

Supplementary Table S7 **Page 21**
Results from logistic regression model to identify host factors associated with unhealthy microbiome pattern for Generation R Study

Supplementary Table S8 **Page 22**
Results from logistic regression model to identify host factors associated with unhealthy microbiome pattern for Rotterdam Study

Supplementary Table S9 **Page 23**
Results from logistic regression model to identify host factors associated with unhealthy microbiome pattern for Lifelines-DEEP Study

Supplementary Table S10 **Page 24**
2x2 contingency table for the association between unhealthy metabolic profiles and unhealthy gut microbiome profiles

Supplementary Table S11 **Page 25**
Risk of atherosclerotic cardiovascular disease (ASCVD) between two microbiome clusters

Supplementary Table S12 **Page 26**
Univariate comparison of metabolic clusters classified by K-Means clustering based on host metabolic phenotypes within Generation R Study, Rotterdam Study and Lifelines-DEEP Study.

Figure S1 **Page 29**
Overlapped cluster-driving genera between Rotterdam Study and Generation R Study.

Figure S2 **Page 30**
Overlapped cluster-driving genera between Rotterdam Study and Lifelines-DEEP Study.

Figure S3 **Page 31**
Overlapped cluster-driving genera between Generation R Study and Lifelines-DEEP Study.

Figure S4 **Page 32**
Two-dimensional representation of the metabolic phenotypes-based clustering of (a) Generation R Study, (b) Rotterdam Study, and (c) Lifelines-DEEP Study.

Reference **Page 33**

Supplementary Material: eMethods

Sample collection and processing

Details of stool sample collection for RS and GenR can be found elsewhere.[1] Briefly, participants of both RS and GenR sent stool samples collected at home to Erasmus MC Rotterdam through regular mail. Participants (or participant's parents or caregivers for GenR) also filled in a short questionnaire including the date and time of defecation and current or recent antibiotic use (past year). Samples that took longer than seven days from production to arrival at the research center (hereafter, TimeInMail) were excluded. To isolate bacterial DNA from stool, an automated stool DNA isolation kit (Diasorin, Saluggia, Italy) was used. The V3 and V4 hypervariable regions of the bacterial 16S rRNA gene were amplified and sequenced on the Illumina MiSeq platform. For LLD, the details of the collection and processing of stool samples are described elsewhere.[2] Briefly, the stool samples were retrieved from participants' homes by students at the University Medical Center Groningen. DNA was isolated with the AllPrep DNA/RNA Mini Kit (Qiagen). The V4 hypervariable region of the bacterial 16S rRNA gene was amplified and sequenced on the Illumina MiSeq platform.

16S rRNA sequencing data profiling

RS and GenR data were processed using the same bioinformatic pipeline for microbiome profiling. For raw reads processing, barcodes were separated from the reads and both ends were pasted together. Reads were then cleaned of primer sequences and heterogeneity spacers using tag cleaner version 0.16.[3] Trimmed reads were then imported within the DADA2 R package version 1.18.0 [4] for microbiome profiling using default parameter settings. A naive Bayesian classifier from the Ribosomal Database Project (RDP2.12), and SILVA 16S database release 138.1 were used to reconstruct the taxonomic composition of the studied communities.[5,6] Amplicon sequence variant (ASV) filtering was applied, i.e., each ASV had to comprise at least 0·5% of the total reads and be present in at least 1% of the study samples to remain in the dataset. Duplicate samples were filtered out as well as swaps and samples with low quality (less than 4·5K reads after the DADA2 pipeline). Taxonomical classification from kingdom through genus was assigned if the bootstrap confidence score was above 50 (i.e., binning posterior probability cut-off was 0·5). Species assignment was only reported if the ASV could be matched to a species with a confidence score of binning posterior probability of 100.

Details of the profiling pipeline applied to samples from LLD are described elsewhere.[7] Briefly, a direct classification of 16S sequencing reads using a naïve Bayesian classifier from the Ribosomal Database Project (RDP2.12), and SILVA 16S database release 128 was used to reconstruct the taxonomic composition of studied communities with a binning posterior possibility cutoff of 0·8. Libraries were rarefied to 10,000 reads prior to taxonomy binning. Furthermore, index sequences were removed and concatenated to generate a unique index of 24bp for each read pair. Paired reads were merged using PEAR with the following settings: minimum overlap of 10 bp and an average read quality phred-score of 20 over a 30 bp sliding window. Merged reads shorter than 200 bp were discarded. Moreover, for extra quality filtering, merged reads that were truncated before three consecutive low-quality bases, ambiguous reads, and chimeric reads were also removed.

Furthermore, for all downstream analyses, an ASV cut-off of 10% prevalence was applied in each cohort independently to reduce data sparsity and computation complexity and increase the probability of replication across cohorts.

Assessment of metabolic phenotypes and covariates

Anthropometric measurements.

The Generation R Study (GenR)

Height (cm) and weight (kg) were measured at the research center in a standing position without shoes and heavy clothing. BMI (kg/m^2) was derived from these measurements. Total body fat mass was measured using DXA (iDXA, GE-Lunar, 2008, Madison, WI, USA), and analyzed with the enCORE software v.12.6. Total body fat percentage was calculated as total body fat mass (kg) divided by body weight (kg). BMI was categorized as underweight, normal BMI, overweight and obesity based on the sex-specific extended International Obesity Task Force (IOTF) cut-offs.[8] In our study population (median age 9·7 years), we identified cut-off points for underweight as below: 14·53 kg/m^2 for boys and 14·46 kg/m^2 for girls; for overweight as over 19·55 kg/m^2 for boys and 19·51 kg/m^2 for girls; and for obesity as over 23·55 kg/m^2 for boys and 23·53 kg/m^2 for girls.

The Rotterdam Study (RS)

Height (cm) and weight (kg) were measured at the research center in a standing position without shoes and heavy clothing. Based on these measurements body mass index (BMI in kg/m^2) was then calculated. Definitions of overweight and obesity were based on WHO classification: overweight: BMI greater than or equal to 25; and obesity: BMI greater than or equal to 30.[9] Waist circumference (WC) was measured midway between the lower rib margin and the iliac crest. Hip circumference (HC) was measured as the distance around the largest part of the hips. The waist-to-hip ratio (WHR) was calculated by dividing WC by HC. Body fat was assessed by dual-energy x-ray absorptiometry (DXA) (GE Lunar Corp, Madison, WI, USA) following manufacturer protocols, with scans analyzed with the enCORE software V13.6.[10] Total body fat percentage was obtained by dividing total body fat mass by weight.

The Lifelines-DEEP Study (LLD)

Height and weight were measured in the research center of University Medical Centre Groningen, and BMI (kg/m^2) was calculated. Definitions of overweight and obesity were based on the WHO classification.[9]

Assessment of blood biochemistry parameters.

The Generation R Study (GenR)

Blood was withdrawn after overnight fasting at the same follow-up visit as stool sampling. Serum glucose was directly measured with the HemoCue® Hb 201 DM System, while insulin was measured with an electrochemiluminescence immunoassay (ECLIA) on the E411 module (Roche, Almere, the Netherlands).[11] A Cobas 8000 analyzer was used to measure serum TC, HDL-c, and triglycerides concentrations with enzymatic essays, whereas C-reactive protein (CRP) was measured by an immuno-turbidimetric assay. Concentrations of insulin and triglycerides were log-transformed to obtain a normal distribution.

The Rotterdam Study (RS)

All biochemical measurements were assessed in serum samples collected after overnight fasting at the same follow-up visit as stool sampling. Serum glucose concentration (mmol/L) was measured using the glucose hexokinase method, and insulin concentration (mmol/L) by an electrochemiluminescence immunoassay. Insulin resistance was calculated using the Homeostatic Model Assessment of Insulin Resistance (HOMA-IR) as follows: fasting insulin (mU/L) × fasting glucose (mmol/L)/22·5.[12] Serum total cholesterol (TC) (mmol/L), high-density lipoprotein-cholesterol (HDL-c) (mmol/L), and triglycerides were measured on the COBAS 8000 Modular Analyzer (Roche Diagnostics, Germany). Concentrations of insulin and triglycerides were log-transformed to obtain a normal distribution.

The Lifelines-DEEP Study (LLD)

Blood was withdrawn at the same follow-up visit as stool sampling. Glucose levels were measured by hydrogen one nuclear magnetic resonance. TC was measured with an enzymatic colorimetric method, HDL_c with a

colorimetric method, and triglycerides with a colorimetric UV method (Modular P analyzer, Roche Diagnostics, Burgdorf, Switzerland).

Measurement of blood pressure.

The Generation R Study (GenR)

When children visited the research facility, we measured their blood pressure at the right brachial artery four times at one-minute intervals using the validated automatic sphygmomanometer Datascope Accutorr Plus (Paramus, New Jersey, United States). For children with complete data on blood pressure, the mean systolic blood pressure (SBP) and diastolic blood pressure (DBP) were calculated from the last three measurements.[13]

The Rotterdam Study (RS)

Blood pressure was measured twice at the right brachial artery with the participant in a sitting position. Hypertension was defined as a mean SBP ≥140mm Hg, a mean DBP ≥ 90mm Hg or a prescription for blood pressure-lowering medications. Drugs categorized by the World Health Organization Anatomical Therapeutic Chemical (WHO ATC) classification [14] as antihypertensives, diuretics, beta-blockers, calcium channel blockers, and RAAS modifying agents were considered blood pressure-lowering medications.

The Lifelines-DEEP Study (LLD)

Blood pressure was measured every minute for ten minutes with an automated DINAMAP Monitor (GE Healthcare, Freiburg, Germany). The size of the cuff was chosen based on the arm circumference of the participant. The average of the final three readings was recorded for SBP and DBP

Assessment of T2D.

The Rotterdam Study (RS)

Cases of T2D were identified as follows: fasting glucose > 6·9mmol/L, non-fasting glucose >11·0 mmol/L, or the use of antidiabetic medication or insulin in the Rotterdam Study. Lab values are obtained at the Rotterdam Study research center as well as from the medical files including general practitioner files and hospital discharge letters of the participants. Two study physicians independently determined all potential events of T2D. In case of disagreement, consensus was sought from an endocrinologist.[15]

The Lifelines-DEEP Study (LLD)

Information on T2D was collected through self-reported questionnaires.

Assessment of ASCVD in the Rotterdam Study (RS)

Incident ASCVD was defined as a composite outcome comprising fatal or nonfatal myocardial infarction, revascularization (including percutaneous coronary intervention and coronary artery bypass grafting), or stroke. Data were obtained through continuous monitoring of medical files from various sources, including general practitioners, hospitals, outpatient clinics, nursing homes, municipal authorities, and national hospital discharge registries. The methods of event adjudication and definitions have been extensively described previously.[16] Each diagnosis underwent validation by two independent research physicians, with consultation from medical specialists in cases of disagreement. The follow-up for ASCVD was defined as the time interval from the stool collection date until the occurrence of the ASCVD, death, loss to follow-up, or January 1st, 2020, whichever happened first.

Assessment of covariates.

The Generation R Study (GenR)

In our association analyses covariates included, ethnicity, maternal education, physical activity, dietary intake and technical covariates. Ethnicity definition followed the classification of Statistics Netherlands, which gives precedence to the birthplace of the mother to assign the ethnicity of the child.[17] Therefore, hereafter ethnicity will be relabeled as migration background. In this study, migration background was re-classified into four main migration background groups: 1) European: including Dutch, Turkish, North African, Oceanic, American and other European; 2) Asian: including Surinamese-Hindu, Indonesian and other Asian; 3) African: including Surinamese-Creole, Sub-Saharan African, Cape Verdean, Antillean, Moroccan and other African; and 4) others: Surinamese unspecified, as previously described.[18] Information on maternal education, physical activity, and dietary intake was obtained through interviews and questionnaires. Maternal education was registered 5 years before the stool collection and re-defined as low (no education, primary education), medium (vocational training or intermediate general school) and high (bachelor's degree or higher academic education) similar to a previous publication.[19] The generation of physical activity scores was adapted from the validated Short QUestionnaire to ASsess Health enhancing physical activity (SQUASH). Parents filled in questionnaires about time (hours per day and days per week) spent outdoors, playing sports and walking/cycling to school.[20] These items were combined into hours per week. Diet was assessed one year before stool sampling by means of a diet quality score.[21] Briefly, the score was based on Dutch dietary guidelines, and contingent on ten food groups (score 0–10) as follows: fruit (≥ 150 g/day), vegetables (≥ 150 g/day), whole grains (≥ 90 g/day), fish (≥ 60 g/week), legumes (≥ 84 g/week), nuts (≥ 15 g/day), dairy (≥ 300 g/day), oils and soft or liquid fats (≥ 30 g/day), sugar-containing beverages (≤ 150 g/day) and high-fat and processed meat (≤ 250 g/week).

Important technical covariates affecting microbiome data were considered in all statistical models. Sampling season was defined as 1) Spring: March-May; 2) Summer: June-August; 3) Autumn: September-November; and 4) Winter: December- February. Both TimeInMail, previously described, and deposition season were obtained from the questionnaires filled by participants during sampling. DNA isolation batch was also considered.[1] Sequencing of samples was originally carried out in 2017;[1] however, an attempt to recover low-quality samples with an improved pipeline was carried out in 2020, and therefore, the sequencing batch was defined on sequencing at these different stages and included as covariate.

The Rotterdam Study (RS)

Information on physical activity, education level, smoking status, diet and alcohol intake was obtained through interviews and questionnaires. All information was collected at the same visit as stool sampling, except for information on diet (including alcohol content), which was obtained about 5 years before stool sampling. Physical activity was assessed with the LASA Physical Activity Questionnaire, and activities were weighted by their intensity with Metabolic Equivalent of Task (MET) as used in previous publication [22] and expressed in MET-hours per week. MET-hours per week were normalized by z-score transformation. Education level was categorized as low (primary, unfinished secondary and lower vocational), medium (secondary or intermediate vocational) or high (higher vocational or university). For smoking, participants were classified as current or non-current smokers. We used a 389-item food frequency questionnaire [23] to determine energy and alcohol intake. Moreover, a score was generated measuring the participants' adherence (yes/no) to 14 items of the Dutch dietary guidelines, with a higher score reflecting better diet quality.[24] Alcohol consumption was converted to a universal unit (g/day, where ten grams of alcohol equals one glass). Information on medication use (Proton pump inhibitors (PPIs) and lipids lowering drugs) was obtained from general practitioners, pharmacies' databases, nationwide medical registries, or follow-up examinations.

Identical technical covariates as those described in relation to the Generation R Study were included in the models (i.e., TimeInMail, sampling season, DNA isolation batch and sequencing batch).

The Lifelines-DEEP Study (LLD)

Information on education, smoking status, dietary intake, and physical activity was assessed through interviews and questionnaires. Physical activity was quantified to a physical activity composition score per week with the Short Questionnaire to assess Health-enhancing physical activity.[25] Total energy intake and alcohol intake were derived from a 125-item food questionnaire.[26] Information on medication use was obtained from general practitioners, pharmacies' databases, Nationwide Medical Registry, or follow-up examinations.

Data Analysis

Missing value handling

For RS and GenR, missing values in covariates were imputed with the mice package in R [8] under the fully conditioned specification. We created ten imputed data sets and pooled results from these analyses using Rubin's rules. For LLD, no imputation was performed.

Microbiome pattern classification

K-Means clustering was performed at the ASV level within each cohort to discover microbiome patterns. Briefly, the ASV table (without rarefaction) was transformed using a center-log-ratio (CLR) function (a pseudo-count of 1 was added to each ASV count before log-transformation), to account for the compositional nature of the microbiome data.[27] The clustering was based on Aitchison's distances across samples using the *kmean* function from the stats package in R. To ensure clustering results were reproducible, three different random seeds were used for each clustering to ensure results are replicable. The consensus of three indexes was used to determine the optimal number of clusters: the Silhouette index, the S_Dbw index, and the Gap statistic index. Clustering results were visualized using the *fviz_cluster* function from the *factoextra* R package, which performs principal component analysis (PCA) and plots the data points according to the first two principal components that explain the largest proportion of the data variance.

Comparison of metabolic phenotypes between microbiome clusters

For continuous variables, a univariate comparison of metabolic phenotypes between clusters was performed using the student's t-test unless a serious deviation from normality was detected, in which the Wilcoxon signed-rank test was used. For categorical variables, a chi-squared test was used.

Identification of genera contributing to cluster assignment

Differential abundant (DA) taxa at the genus level were identified by ANCOM-BC, which estimates the unknown sampling fractions and corrects the bias induced by these differences among samples.[28] Before analysis, taxa were aggregated at the genus level with *tax_glom* function from the R *phyloseq* package.[29] Default parameters were used for both RS and GenR cohorts. Models were adjusted for potential confounders: for RS, these included age, sex, BMI, smoking, alcohol intake, physical activity, energy intake, dietary quality score, education level, lipids lowering drug use, PPIs use, and technical covariates (i.e., TimeInMail, sampling season, DNA isolation batch, and sequencing batch); for GenR, these included age, sex, BMI, ethnicity, physical activity, energy intake, dietary quality score, maternal education level, and the above-mentioned technical covariates; for LLD, these included age, sex, BMI, smoking, alcohol intake, physical activity, energy intake, education level, lipids lowering drug use, and PPIs use. After analysis, Bonferroni correction was used to account for multiple testing.

Identification of host risk factors associated with microbiome cluster assignment

Host risk factors associated with microbiome cluster assignment were identified with a logistic regression. We started with a saturated model where all metabolic phenotypes and possible confounders were included. For RS, the initial model included age, sex, obesity, body fat percentage, triglycerides, HDL_c, glucose, insulin, mean SBP, mean DBP, physical activity, energy intake, diet quality score, smoking, alcohol intake, medication use (i.e., PPIs and lipid-lowering medications), education level, and technical covariates. For GenR, the initial model included age, sex, ethnicity, obesity, body fat percentage, triglycerides, HDL_c, glucose, insulin, mean SBP, mean DBP, physical activity, energy intake, diet quality score, maternal education level and technical covariates. For LLD, the initial model included age, sex, obesity, triglycerides, HDL_c, glucose, mean SBP, mean DBP, physical activity, energy intake, smoking, alcohol intake, medication use (i.e., PPIs and lipid-lowering medications), and education level. For all models, reference for sex is female; for RS and LLD, reference for medication use is non-use, for smoking is non-smoking; for RS and GenR, reference for season is Spring, for SeqBatch is 0, for DNA isolation batch is 0; for GenR, reference of ethnicity is European. Reference for education (or maternal education) is low in RS and GenR, while medium is used as reference in LLD. This is because the number of individuals with a low level of education is extremely small in LLD. For all models, collinearity was checked, and variables with a variance inflation factor (VIF) >10 were excluded. We then used backward selection to identify significant associations with the *stepAIC* function from the R *MASS* package. This works by removing one predictor each time based on model AIC value calculation. The goal is to identify which variable, when removed, leads to the least loss of model performance relative to the complexity added. This iterative process will continue until no further reduction in AIC can be achieved by removing additional variables. The best model was selected as final model.

Host metabolic healthy status classification

To determine individual metabolic-healthy status, a K-Means cluster analysis was performed on host metabolic phenotype data. For RS, the input features for the K-Means algorithm included age, BMI, WHR, body fat percentage, glucose, insulin, TC, HDL_c, triglycerides, mean SBP, and mean DBP. For GenR, these included age, BMI, body fat percentage, glucose, insulin, TC, HDL_c, triglycerides, mean SBP, mean DBP and CRP. For LLD, these included age, BMI, WHR, glucose, TC, HDL_c, triglycerides, mean SBP, and mean DBP. All features were standardized by central scaling, and differences in the overall metabolic profiles between two participants were measured with Euclidean distances. For each cohort, the optimal number of clusters was determined based on the consensus of three indexes as previously described for the definition of microbiome-based clusters.

Association between microbiome cluster assignment and host metabolic-healthy status

For all three cohorts, the association between microbiome cluster assignment and metabolic-healthy status was assessed by the odds ratio derived from a contingency table.

Sample inclusion and exclusion for GenR and RS

For GenR, excluded samples include: 1) individuals with antibiotic use within one month before stool production (n=13) or without antibiotic use data (n=214), 2) the missing values either for TimeInMail (n=22), ethnicity (n=32), BMI (n=1), obesity (n=6), body fat percentage (n=14), and blood pressure (n=42) and participants without inform of consent (n=649).

For RS, excluded samples include: 1) participants who used antibiotics within one month before stool collection (n=49) or didn't have antibiotic use data (n=14), 2) participants with missing values either for sample mailing time (i.e., TimeInMail, n=30) or any of the outcome variables (n=40), 3) participants for whom blood and stool were collected more than six months apart (n=11) or for whom such information was missing (n=12). As a result, samples from 1,265 participants were included in the analyses.

Supplementary Tables

Table S1. An overview of metabolic phenotypes and covariates data available per cohort, and variables used for each step analysis.

	GenR	RS	LLD
**-Metabolic phenotypes**			
Age	√, X,$, #	√, X, $, #	√, X,$, #
Sex	√, X,$	√, X, $	√, X,$
BMI	√,X, #	√, X, #	√, X, #
Body fat percent	√,$, #	√, $, #	
Waist-to-hip ratio		√, #	√, #
Obesity status	√,$	√, $	√,$
Glucose	√,$, #	√,$, #	√,$, #
Insulin	√,$, #	√,$, #	
Insulin resistance		√	
Total cholesterol	√, #	√, #	√, #
HDL-c	√,$, #	√,$, #	√,$, #
Triglycerides	√,$, #	√,$, #	√,$, #
C-reactive protein	√, #		√
Mean systolic blood pressure (SBP)	√,$, #	√,$,#	√,$, #
Mean diastolic blood pressure (DBP)	√,$, #	√,$,#	√,$, #
T2D		√	√
Hypertension		√	√
ASCVD		√	
**-Covariates**			
Ethnicity	√, X,$		
Physical activity	√, X,$	√, X,$	√, X,$
Education/Maternal education	√, X,$	√, X,$	√, X,$
Smoking		√, X,$	√, X,$
Alcohol intake		√, X,$	√, X,$
Energy intake	√, X,$	√, X,$	√, X,$
Diet quality score	√, X,$	√, X,$	
PPIs		√, X,$	√, X,$
Lipids lowering drugs		√, X,$	√, X,$
TimeInMail	√, X,$	√, X,$	
Season	√, X,$	√, X,$	
DNA isolation batch	√, X,$	√, X,$	
Sequencing batch	√, X,$	√, X,$	

"√": data available.
"X": used for covariates in differential abundant taxa identification.
"$": used in initial model for identification of host factors associated with unhealthy microbiome pattern.
"#": used for host metabolic health status clustering.

Table S2. Best cluster number determination for microbiome-ASV based clustering based on three different algorithms*

**Cohort**	**Algorithm**	Random seed: *1234*		Random seed: *2345*		Random seed: *3456*	
		Index value	Best cluster number	Index value	Best cluster number	Index value	Best cluster number
**GenR**	Silhouette index	0.04	2	0.04	2	0.04	2
	S_Dbw index	0.94	17	0.94	17	0.94	17
	Gap statistic index	-0.68	2	-0.68	2	-0.68	2
	**Optimal cluster number**	--	**2**	--	**2**	--	**2**
**RS**	Silhouette index	0.04	2	0.04	2	0.04	2
	S_Dbw index	0.94	34	0.94	34	0.94	34
	Gap statistic index	-0.61	2	-0.61	2	-0.61	2
	**Optimal cluster number**	--	**2**	--	**2**	--	**2**
**LLD**	Silhouette index	0.41	2	0.41	2	0.41	2
	S_Dbw index	0.92	37	0.92	37	0.92	37
	Gap statistic index	-0.68	2	-0.68	2	-0.68	2
	**Optimal cluster number**	--	**2**	--	**2**	--	**2**

"*": Cluster number range was defined between 2 to 50

Table S3. Best cluster number determination for metabolic phenotype data clustering based on three different algorithms*

**Cohort**	**Algorithm**	Random seed: *1234*		Random seed: *2345*		Random seed: *3456*	
		Index value	Best cluster number	Index value	Best cluster number	Index value	Best cluster number
**GenR**	Silhouette index	0.17	2	0.17	2	0.17	2
	S_Dbw index	0.45	50	0.45	50	0.45	50
	Gap statistic index	0.62	2	0.62	2	0.62	2
	**Optimal cluster number**	--	**2**	--	**2**	--	**2**
**RS**	Silhouette index	0.17	2	0.17	2	0.17	2
	S_Dbw index	0.42	50	0.42	50	0.42	50
	Gap statistic index	0.99	2	0.99	2	0.99	2
	**Optimal cluster number**	--	**2**	--	**2**	--	**2**
**LLD**	Silhouette index	0.22	2	0.22	2	0.22	2
	S_Dbw index	1.26	2	1.26	2	1.26	2
	Gap statistic index	0.80	2	0.80	2	0.80	2
	**Optimal cluster number**	--	**2**	--	**2**	--	**2**

"*": Cluster number range was defined between 2 to 50

Table S4. Differential abundant taxa between clusters of Generation R Study identified by ANCOM-BC

Taxa	beta.clusterU	se.clusterU	p_val	q_val	diff_abn
Agathobacter	0.1	0.09	0.28	1.00	FALSE
Escherichia-Shigella	-0.16	0.18	0.38	1.00	FALSE
Faecalibacterium	-0.29	0.07	1.13E-04	3.15E-03	TRUE
Bacteroides	-0.36	0.08	3.68E-06	1.18E-04	TRUE
Subdoligranulum	0.49	0.08	2.67E-10	1.15E-08	TRUE
Alistipes	-0.8	0.1	5.64E-17	3.1E-15	TRUE
Dialister	0.01	0.17	0.96	1.00	FALSE
Oscillospiraceae UCG-002	-1.19	0.09	1.40E-40	1.03E-38	TRUE
Blautia	0.95	0.06	2.63E-53	2.07E-51	TRUE
Fusicatenibacter	1.01	0.09	5.68E-32	4.03E-30	TRUE
Christensenellaceae R-7 group	-0.75	0.12	2.00E-10	8.79E-09	TRUE
Bifidobacterium	1.44	0.1	3.95E-44	3.00E-42	TRUE
Romboutsia	1.45	0.11	1.65E-38	1.20E-36	TRUE
Parabacteroides	-0.98	0.12	1.03E-16	5.55E-15	TRUE
Prevotella_9	-1.72	0.2	3.38E-18	1.93E-16	TRUE
Roseburia	0.01	0.11	0.929251	1.00	FALSE
[Eubacterium] hallii group	1.45	0.09	4.76E-61	3.81E-59	TRUE
Dorea	1.22	0.09	7.73E-47	5.95E-45	TRUE
Coprococcus	0.02	0.09	0.78	1.00	FALSE
Lachnospiraceae NK4A136 group	-0.71	0.09	4.02E-14	2.01E-12	TRUE
Ruminococcus	-0.01	0.11	0.91	1.00	FALSE
Anaerostipes	1.18	0.09	2.22E-43	1.67E-41	TRUE
[Eubacterium] eligens group	-0.67	0.12	4.29E-08	1.71E-06	TRUE
[Ruminococcus] torques group	1.01	0.1	1.74E-25	1.10E-23	TRUE
CAG-352	-0.52	0.13	1.07E-04	0.003117	TRUE
[Eubacterium] siraeum group	-1.35	0.12	6.45E-28	4.19E-26	TRUE
Phascolarctobacterium	-0.64	0.14	3.05E-06	1.01E-04	TRUE
NK4A214 group	-0.68	0.12	4.40E-09	1.85E-07	TRUE
[Eubacterium] ruminantium group	-1.59	0.14	1.75E-31	1.22E-29	TRUE
Lachnospira	-0.41	0.12	1.17E-03	0.03	TRUE
Intestinibacter	1.33	0.12	4.43E-29	3.01E-27	TRUE
Clostridium sensu stricto 1	1.29	0.12	1.21E-25	7.72E-24	TRUE
Oscillospiraceae UCG-005	-1.24	0.11	8.27E-29	5.46E-27	TRUE
Lachnoclostridium	-0.09	0.11	0.44	1.00	FALSE
[Eubacterium] ventriosum group	0.07	0.12	0.59	1.00	FALSE
Lachnospiraceae ND3007 group	0.26	0.11	0.02	0.42	FALSE
Akkermansia	-0.22	0.12	0.07	1.00	FALSE
Monoglobus	0.53	0.1	1.40E-07	5.30E-06	TRUE
Erysipelotrichaceae UCG-003	1.06	0.11	2.00E-23	1.24E-21	TRUE
[Ruminococcus] gauvreauii group	1.11	0.12	4.86E-22	2.92E-20	TRUE
Odoribacter	-1.27	0.11	3.18E-31	2.19E-29	TRUE

Incertae Sedis	0.92	0.09	4.07E-23	2.48E-21	TRUE
[Eubacterium] xylanophilum group	-1	0.12	2.36E-16	1.25E-14	TRUE
Oscillospiraceae UCG-003	-1.53	0.11	2.88E-47	2.25E-45	TRUE
Streptococcus	1.35	0.12	7.10E-29	4.75E-27	TRUE
UBA1819	0.65	0.11	4.78E-09	1.96E-07	TRUE
Paraprevotella	-0.92	0.13	5.37E-12	2.47E-10	TRUE
CAG-56	0.01	0.12	0.94	1.00	FALSE
Butyricicoccus	0.02	0.11	0.88	1.00	FALSE
Haemophilus	-0.07	0.12	0.53	1.00	FALSE
Lachnospiraceae UCG-004	-1.16	0.12	7.02E-22	4.14E-20	TRUE
Terrisporobacter	0.57	0.11	4.68E-07	1.68E-05	TRUE
Lachnospiraceae FCS020 group	0.76	0.1	4.26E-14	2.09E-12	TRUE
Turicibacter	0.45	0.1	1.18E-05	3.54E-04	TRUE
Barnesiella	-0.59	0.12	1.06E-06	3.72E-05	TRUE
Lachnospiraceae AC2044 group	-1.18	0.1	2.30E-33	1.66E-31	TRUE
[Ruminococcus] gnavus group	0.28	0.11	8.70E-03	0.19	FALSE
Lachnospiraceae UCG-010	-0.86	0.11	3.00E-16	1.56E-14	TRUE
Family XIII AD3011 group	0.53	0.1	8.41E-08	3.28E-06	TRUE
Sutterella	-0.86	0.12	2.09E-12	9.81E-11	TRUE
Oscillibacter	-0.24	0.1	0.02	0.46	FALSE
Hungatella	-0.15	0.1	0.13	1.00	FALSE
Parasutterella	-0.45	0.09	1.87E-06	6.37E-05	TRUE
Bilophila	-0.64	0.1	1.70E-11	7.67E-10	TRUE
Family XIII UCG-001	-0.29	0.1	2.46E-03	0.06	FALSE
Colidextribacter	-0.82	0.1	5.21E-16	2.66E-14	TRUE
Lachnospiraceae UCG-001	-0.42	0.1	1.01E-05	3.12E-04	TRUE
GCA-900066575	-0.87	0.09	1.32E-21	7.68E-20	TRUE
Collinsella	0.01	0.09	0.94	1.00	FALSE
Lachnospiraceae UCG-003	-0.61	0.09	7.41E-13	3.56E-11	TRUE
Tyzzerella	-0.27	0.09	2.14E-03	0.053	FALSE
Flavonifractor	0.19	0.09	0.04	0.81	FALSE
DTU089	-0.01	0.09	0.94	1.00	FALSE
Marvinbryantia	0.04	0.08	0.63	1.00	FALSE
Veillonella	0.02	0.1	0.84	1.00	FALSE
Paludicola	-0.42	0.08	3.96E-07	1.46E-05	TRUE
Coprobacillus	-0.25	0.08	1.98E-03	0.051365	FALSE
Oscillospira	-0.6	0.07	1.63E-17	9.12E-16	TRUE
Anaerotruncus	-0.2	0.07	5.40E-03	0.12	FALSE
Erysipelatoclostridium	-0.07	0.07	0.35	1.00	FALSE

"beta.clusterU"=Effect size for clusterU

"se.clusterU"= Standard error for effect size of clusterU

"p_val"= P value

"q_val"= q value (or adjusted p value)

"diff_abn"=Logistic value for if this taxa is differential abundant or not

Table S5. Differential abundant taxa between clusters of Rotterdam Study identified by ANCOM-BC

Taxa	beta.clusterU	se.clusterU	p_val	q_val	diff_abn
Blautia	0.87	0.07	4.42E-35	4.11E-33	TRUE
Agathobacter	0.41	0.11	1.78E-04	8.72E-03	TRUE
Escherichia-Shigella	-1.00	0.20	3.11E-07	1.93E-05	TRUE
Faecalibacterium	-0.20	0.10	0.04	1.00	FALSE
[Eubacterium] hallii group	0.56	0.10	5.89E-08	3.89E-06	TRUE
Fusicatenibacter	0.46	0.11	5.35E-05	2.78E-03	TRUE
Subdoligranulum	0.05	0.10	0.65	1.00	FALSE
Dorea	0.49	0.10	6.48E-07	3.95E-05	TRUE
Anaerostipes	0.95	0.10	5.7E-21	4.79E-19	TRUE
Romboutsia	0.00	0.16	0.97	1.00	FALSE
Coprococcus	-0.53	0.10	1.03E-07	6.58E-06	TRUE
Bifidobacterium	0.45	0.17	8.58E-03	0.34	FALSE
Bacteroides	-0.22	0.11	0.04	1.00	FALSE
Ruminococcus	-0.14	0.15	0.34	1.00	FALSE
Oscillospiraceae UCG-002	-2.01	0.11	4.65E-74	4.51E-72	TRUE
[Ruminococcus] torques group	0.64	0.11	1.85E-09	1.28E-07	TRUE
CAG-352	-1.01	0.18	1.97E-08	1.32E-06	TRUE
Streptococcus	1.64	0.15	6.1E-29	5.49E-27	TRUE
Intestinibacter	0.19	0.17	0.26	1.00	FALSE
Christensenellaceae R-7 group	-1.91	0.12	1.36E-59	1.29E-57	TRUE
Lachnospiraceae ND3007 group	-0.31	0.12	7.14E-03	0.30	FALSE
Roseburia	0.13	0.10	0.19	1.00	FALSE
[Ruminococcus] gauvreauii group	-0.06	0.15	0.71	1.00	FALSE
Clostridium sensu stricto 1	-0.41	0.16	0.01	0.44	FALSE
Dialister	1.04	0.16	1.35E-10	9.69E-09	TRUE
[Eubacterium] siraeum group	-1.57	0.14	1.09E-27	9.55E-26	TRUE
Butyrivibrio	-1.32	0.14	2.15E-21	1.83E-19	TRUE
Lachnospiraceae NK4A136 group	-0.92	0.12	3.76E-15	3.05E-13	TRUE
[Eubacterium] ruminantium group	-1.60	0.16	1.85E-24	1.61E-22	TRUE
Alistipes	-0.41	0.12	7.10E-04	0.03	TRUE
Erysipelotrichaceae UCG-003	0.78	0.14	6.21E-08	4.04E-06	TRUE
[Eubacterium] eligens group	-0.93	0.14	2.73E-11	2.08E-09	TRUE
Monoglobus	0.74	0.11	2.89E-12	2.22E-10	TRUE
Parabacteroides	-0.61	0.14	8.00E-06	4.56E-04	TRUE
Akkermansia	-0.20	0.14	0.16	1.00	FALSE
Incertae Sedis	-0.03	0.10	0.76	1.00	FALSE
Lachnospira	-0.52	0.13	1.01E-04	5.04E-03	TRUE
Prevotella_9	-0.58	0.14	4.33E-05	2.30E-03	TRUE
Butyricicoccus	0.53	0.12	8.11E-06	4.56E-04	TRUE
CAG-56	-0.08	0.14	0.56	1.00	FALSE

Lachnoclostridium	0.24	0.12	0.05	1.00	FALSE
[Eubacterium] ventriosum group	0.16	0.12	0.19	1.00	FALSE
Terrisporobacter	-0.23	0.14	0.09	1.00	FALSE
NK4A214 group	-2.00	0.12	1.50E-62	1.44E-60	TRUE
[Ruminococcus] gnavus group	1.46	0.13	6.34E-29	5.65E-27	TRUE
Phascolarctobacterium	-0.18	0.14	0.21	1.00	FALSE
Holdemanella	-0.56	0.14	7.79E-05	3.98E-03	TRUE
[Eubacterium] xylanophilum group	-1.22	0.13	7.53E-22	6.47E-20	TRUE
Oscillospiraceae UCG-005	-2.02	0.10	2.20E-82	2.16E-80	TRUE
Lachnospiraceae FCS020 group	0.19	0.12	0.11	1.00	FALSE
Turicibacter	-0.15	0.14	0.27	1.00	FALSE
UBA1819	0.81	0.12	1.93E-12	1.50E-10	TRUE
Collinsella	0.25	0.13	0.05	1.00	FALSE
Paraprevotella	-0.89	0.14	4.91E-11	3.68E-09	TRUE
Marvinbryantia	-0.62	0.13	1.21E-06	7.17E-05	TRUE
Intestinimonas	-0.90	0.12	1.99E-13	1.57E-11	TRUE
Oscillospiraceae UCG-003	-1.58	0.12	1.55E-42	1.46E-40	TRUE
Tyzzerella	0.65	0.12	1.22E-07	7.68E-06	TRUE
Family XIII AD3011 group	-0.28	0.10	7.62E-03	0.31	FALSE
Lachnospiraceae UCG-001	-0.26	0.12	0.03	1.00	FALSE
Colidextribacter	-1.35	0.11	1.77E-32	1.63E-30	TRUE
Oscillibacter	0.36	0.12	1.74E-03	0.08	FALSE
Lactobacillus	0.14	0.10	0.16	1.00	FALSE
Lachnospiraceae UCG-004	-0.61	0.12	1.01E-06	6.07E-05	TRUE
Lachnospiraceae UCG-010	0.06	0.12	0.59	1.00	FALSE
Lachnospiraceae AC2044 group	-1.22	0.11	2.33E-30	2.12E-28	TRUE
Odoribacter	-0.73	0.12	3.73E-10	2.65E-08	TRUE
Family XIII UCG-001	-0.09	0.12	0.44	1.00	FALSE
Senegalimassilia	-0.09	0.11	0.41	1.00	FALSE
Howardella	-0.43	0.12	3.23E-04	0.02	TRUE
Desulfovibrio	-0.44	0.11	3.16E-05	1.71E-03	TRUE
Sutterella	-0.25	0.11	0.03	1.00	FALSE
Negativibacillus	-0.07	0.11	0.55	1.00	FALSE
DTU089	0.02	0.10	0.82	1.00	FALSE
Parasutterella	-0.05	0.10	0.58	1.00	FALSE
Slackia	0.07	0.09	0.40	1.00	FALSE
Haemophilus	0.16	0.08	0.06	1.00	FALSE
GCA-900066575	-0.62	0.10	7.29E-11	5.40E-09	TRUE
Flavonifractor	0.86	0.09	1.18E-20	9.82E-19	TRUE
Enterorhabdus	-0.06	0.09	0.52	1.00	FALSE
Lactococcus	0.29	0.09	1.59E-03	0.07	FALSE
UC5-1-2E3	0.58	0.09	8.28E-11	6.04E-09	TRUE
Erysipelatoclostridium	0.75	0.09	1.04E-16	8.49E-15	TRUE
Bilophila	0.01	0.09	0.90	1.00	FALSE
Shuttleworthia	-0.14	0.09	0.14	1.00	FALSE

Oscillospira	0.02	0.09	0.83	1.00	FALSE
[Eubacterium] brachy group	0.66	0.09	8.72E-15	6.97E-13	TRUE
Coprobacter	-0.01	0.08	0.90	1.00	FALSE
Butyricimonas	-0.11	0.08	0.18	1.00	FALSE
[Eubacterium] fissicatena group	0.44	0.07	5.07E-09	3.45E-07	TRUE
Paludicola	-0.19	0.08	0.01	0.51	FALSE
Mogibacterium	0.06	0.08	0.42	1.00	FALSE
TM7x	0.46	0.08	1.81E-09	1.26E-07	TRUE
Defluviitaleaceae UCG-011	-0.27	0.07	2.37E-04	0.01	TRUE
UCG-007	-0.22	0.07	3.12E-03	0.13	FALSE
Candidatus Soleaferrea	0.33	0.07	1.70E-06	9.86E-05	TRUE
Holdemania	0.29	0.07	1.46E-05	8.04E-04	TRUE
Butyricicoccaceae UCG-009	0.03	0.07	0.65	1.00	FALSE

"beta.clusterU"=Effect size for clusterU

"se.clusterU"= Standard error for effect size of clusterU

"p_val"= P value

"q_val"= q value (or adjusted p value)

"diff_abn"=Logistic value for if this taxa is differential abundant or not

Table S6. Differential abundant taxa between clusters of Lifelines-DEEP Study identified by ANCOM-BC

Taxa	lfc.clusterU	se.clusterU	p_val	q_val	diff_abn
Bifidobacterium	-0.36	0.25	0.15	1.00	FALSE
Blautia	0.84	0.20	3.28E-05	2.16E-03	TRUE
Ruminococcus_2	-1.41	0.28	3.41E-07	2.86E-05	TRUE
Faecalibacterium	0.47	0.20	0.02	0.80	FALSE
Subdoligranulum	-0.14	0.20	0.50	1.00	FALSE
Romboutsia	-0.63	0.23	5.98E-03	0.29	FALSE
Agathobacter	1.06	0.20	1.12E-07	9.64E-06	TRUE
Dialister	1.20	0.30	9.04E-05	5.70E-03	TRUE
Collinsella	-0.54	0.28	0.05	1.00	FALSE
Fusicatenibacter	0.86	0.19	9.03E-06	6.69E-04	TRUE
Erysipelotrichaceae_UCG-003	0.72	0.23	2.07E-03	0.11	FALSE
Dorea	0.26	0.18	0.14	1.00	FALSE
Streptococcus	1.17	0.20	6.18E-09	5.50E-07	TRUE
Clostridium_sensu_stricto_1	-1.43	0.23	5.93E-10	5.40E-08	TRUE
Intestinibacter	-0.69	0.24	5.02E-03	0.26	FALSE
Akkermansia	-1.23	0.27	4.66E-06	3.64E-04	TRUE
Anaerostipes	0.88	0.19	2.00E-06	1.62E-04	TRUE
Methanobrevibacter	-3.42	0.24	5.43E-45	5.65E-43	TRUE
Bacteroides	0.37	0.20	0.06	1.00	FALSE
Holdemanella	-1.28	0.29	8.82E-06	6.62E-04	TRUE
Coprococcus_3	0.12	0.20	0.55	1.00	FALSE
Christensenellaceae_R-7_group	-1.53	0.19	2.1E-16	2.12E-14	TRUE
Prevotella_9	-1.46	0.26	3.47E-08	3.02E-06	TRUE
Ruminococcus_1	0.07	0.22	0.75	1.00	FALSE
Ruminococcaceae_UCG-013	0.84	0.17	1.46E-06	01.20E-04	TRUE
Catenibacterium	-0.40	0.22	0.07	1.00	FALSE
Ruminococcaceae_UCG-002	-1.48	0.18	9.18E-16	9.18E-14	TRUE
Ruminiclostridium_5	0.13	0.18	0.46	1.00	FALSE
Butyricicoccus	0.46	0.18	0.01	0.49	FALSE
Coprococcus_1	-0.53	0.17	1.87E-03	0.11	FALSE
Turicibacter	-1.09	0.23	3.37E-06	2.69E-04	TRUE
Coprococcus_2	-1.85	0.24	4.16E-15	3.99E-13	TRUE
Alistipes	0.06	0.19	0.74	1.00	FALSE
Lachnospiraceae_ND3007_group	0.10	0.19	0.61	1.00	FALSE
Enterorhabdus	-0.95	0.23	2.52E-05	1.69E-03	TRUE
Parabacteroides	-0.19	0.21	0.37	1.00	FALSE
Senegalimassilia	-0.80	0.23	4.58E-04	0.03	TRUE
Roseburia	0.23	0.20	0.24	1.00	FALSE
Terrisporobacter	-0.98	0.22	9.75E-06	7.12E-04	TRUE
Escherichia/Shigella	0.36	0.21	0.09	1.00	FALSE

Lachnospiraceae_NK4A136_group	0.26	0.20	0.18	1.00	FALSE
CAG-56	0.51	0.21	0.02	0.69	FALSE
Ruminococcaceae_UCG-005	-2.15	0.17	5.11E-35	5.27E-33	TRUE
Phascolarctobacterium	-0.51	0.23	0.03	1.00	FALSE
Intestinimonas	-0.93	0.21	6.96E-06	5.36E-04	TRUE
Ruminococcaceae_UCG-014	-1.77	0.22	3.77E-15	3.65E-13	TRUE
Ruminococcaceae_UCG-004	1.79	0.23	1.97E-14	1.87E-12	TRUE
Ruminococcaceae_NK4A214_group	-1.74	0.19	1.88E-19	1.92E-17	TRUE
Adlercreutzia	1.41	0.21	9.41E-12	8.75E-10	TRUE
Ruminiclostridium_6	-0.87	0.20	1.90E-05	1.29E-03	TRUE
Family_XIII_AD3011_group	-0.70	0.17	4.20E-05	2.73E-03	TRUE
Prevotella_7	0.14	0.19	0.44	1.00	FALSE
Peptococcus	-0.18	0.19	0.35	1.00	FALSE
Ruminococcaceae_UCG-008	-0.55	0.19	2.97E-03	0.16	FALSE
Lactobacillus	0.93	0.20	3.85E-06	3.04E-04	TRUE
Lachnospiraceae_FCS020_group	0.24	0.18	0.18	1.00	FALSE
Ruminococcaceae_UCG-003	-0.81	0.18	1.07E-05	7.60E-04	TRUE
Faecalitalea	0.06	0.19	0.74	1.00	FALSE
Butyrivibrio	-0.62	0.18	3.83E-04	0.02	TRUE
Parasutterella	0.55	0.18	2.84E-03	0.15	FALSE
Paraprevotella	-0.40	0.20	0.05	1.00	FALSE
Erysipelatoclostridium	1.55	0.20	2.61E-15	2.56E-13	TRUE
Mogibacterium	0.00	0.18	0.98	1.00	FALSE
Slackia	-0.19	0.19	0.32	1.00	FALSE
Lachnospira	0.01	0.20	0.95	1.00	FALSE
Lachnospiraceae_UCG-001	0.68	0.20	4.95^{E}-04	0.03	TRUE
Ruminiclostridium_9	-1.12	0.18	2.00E-10	1.84E-08	TRUE
Lachnoclostridium	0.98	0.19	1.57E-07	1.34E-05	TRUE
Eggerthella	1.48	0.18	1.53E-15	1.51E-13	TRUE
Oscillibacter	0.79	0.18	8.61E-06	6.54E-04	TRUE
Family_XIII_UCG-001	-0.32	0.17	0.06	1.00	FALSE
Lachnospiraceae_UCG-010	0.35	0.19	0.06	1.00	FALSE
Lactococcus	0.78	0.18	1.31E-05	9.17E-04	TRUE
Desulfovibrio	-0.17	0.19	0.37	1.00	FALSE
Barnesiella	-0.05	0.17	0.76	1.00	FALSE
Bilophila	0.38	0.18	0.04	1.00	FALSE
Haemophilus	0.16	0.17	0.35	1.00	FALSE
Tyzzerella_3	0.81	0.18	1.04E-05	7.51E-04	TRUE
Lachnospiraceae_UCG-008	-0.47	0.17	5.11E-03	0.26	FALSE
Sutterella	0.03	0.19	0.88	1.00	FALSE
UBA1819	0.54	0.17	1.92E-03	0.11	FALSE
Actinomyces	0.47	0.17	7.20E-03	0.34	FALSE
Marvinbryantia	-0.40	0.18	0.03	1.00	FALSE
Odoribacter	-0.06	0.16	0.71	1.00	FALSE
Herbinix	0.22	0.17	0.20	1.00	FALSE
Negativibacillus	0.38	0.18	0.04	1.00	FALSE

Lachnospiraceae_AC2044_group	-0.23	0.16	0.15	1.00	FALSE
DTU089	0.28	0.17	0.10	1.00	FALSE
Gordonibacter	0.83	0.16	4.01E-07	3.32E-05	TRUE
Ruminococcaceae_UCG-010	-1.04	0.17	1.03E-09	9.26E-08	TRUE
GCA-900066575	0.21	0.16	0.18	1.00	FALSE
Veillonella	0.40	0.16	0.01	0.56	FALSE
Flavonifractor	1.11	0.16	6.17E-12	5.80E-10	TRUE
Howardella	-0.24	0.16	0.14	1.00	FALSE
Leuconostoc	0.54	0.16	7.02E-04	0.04	TRUE
Lachnospiraceae_UCG-004	0.44	0.15	3.97E-03	0.21	FALSE
Allisonella	0.32	0.16	0.04	1.00	FALSE
Merdibacter	0.61	0.15	6.69E-05	4.28E-03	TRUE
Oscillospira	0.42	0.15	6.41E-03	0.31	FALSE
Oxalobacter	-0.10	0.14	0.48	1.00	FALSE
Ruminococcaceae_UCG-009	0.52	0.15	4.95E-04	0.03	TRUE
Holdemania	0.62	0.14	1.77E-05	1.22E-03	TRUE
Gemella	0.82	0.14	1.37E-08	1.21E-06	TRUE
Coprobacter	0.35	0.14	0.01	0.64	FALSE

"beta.clusterU"=Effect size for clusterU

"se.clusterU"= Standard error for effect size of clusterU

"p_val"= P value

"q_val"= q value (or adjusted p value)

"diff_abn"=Logistic value for if this taxa is differential abundant or not

Table S7. Results from logistic regression model to identify host factors associated with unhealthy microbiome pattern for Generation R Study

	[4]**OR**	**95%CI**	**P**
Intercept	0.34	(0.11, 1.03)	0.06
Sex (Male)	1.04	(0.82, 1.31)	0.75
African	0.86	(0.62, 1.20)	0.38
Asian	1.74	(1.11, 2.75)	0.02
Others	1.36	(0.46, 4.05)	0.57
Fat Percent	1.03	(1.01, 1.05)	<0.01
[1]Triglycerides	1.36	(1.04, 1.77)	0.02
HDL_c	1.40	(0.98, 2.01)	0.07
[2]Maternal Education Medium	0.63	(0.34, 1.17)	0.15
Maternal Education High	0.43	(0.23, 0.81)	<0.01
TimeInMail	1.10	(1.02, 1.20)	0.02
[3]DNA Isolation Batch one	7.72	(4.80, 13.04)	<0.001
Season Summer	0.72	(0.53, 0.96)	0.03
Season Autumn	1.06	(0.79, 1.43)	0.68
Season Winter	0.75	(0.56, 1.01)	0.06

"1": Triglycerides is log10-transformed.
"2": Low education level is used as reference.
"3": DNA isolation batch zero is used as reference.
"4": OR= exponential of coefficient.
"*": P value is significant.

Table S8. Results from logistic regression model to identify host factors associated with unhealthy microbiome pattern for Rotterdam Study

	[3]OR	95%CI	P
Intercept	0.15	(0.03,0.72)	0.018*
Sex (Male)	1.49	(1.04, 2.13)	0.03*
Fat Percent	1.05	(1.02, 1.07)	<0.001*
[1]Triglycerides	4.11	(1.96, 8.75)	<0.001*
HDL_c	1.35	(0.96, 1.91)	0.08
Systolic blood pressure	1.01	(1.00, 1.02)	0.15
Dystolic blood pressure	0.99	(0.97, 1.00)	0.12
[2]Education Medium	0.86	(0.64, 1.15)	0.30
Education High	0.69	(0.51, 0.92)	0.01*
Smoking	1.73	(1.20, 2.51)	<0.01*
Diet quality score	0.95	(0.89, 1.01)	0.11
PPIs	1.37	(1.01, 1.86)	0.04*
Lipids lowering drugs	1.31	(1.00, 1.72)	0.05
TimeInMail	1.14	(1.01, 1.29)	0.03*
SeqBatch one	0.66	(0.50, 0.87)	<0.01*

"1": Triglycerides is log10-transformed
"2": Low education level is used as reference
"3": OR= exponential of coefficient.
"*": P value is significant.

Table S9. Results from logistic regression model to identify host factors associated with unhealthy microbiome pattern for Lifelines-DEEP Study

	[3]OR	95%CI	P
Intercept	2.85	(0.78, 10.55)	0.115
Age	0.96	(0.95, 0.98)	<0.001*
Lipids lowering drugs	2.04	(1.00,4.30)	0.055
PPIs	1.46	(0.89, 2.41)	0.141
Obesity	1.76	(1.12, 2.80)	0.015
Systolic blood pressure	1.01	(1.00, 1.02)	0.179
[1]Triglycerides	4.30	(2.24, 8.37)	<0.001*
[2]Education Low	0.34	(0.07, 1.36)	0.145
[2]Education High	0.63	(0.48, 0.83)	0.001*

“1”: Triglycerides is log10-transformed.
“2”: Medium education level is used as reference.
“3”: OR= exponential of coefficient.
“*”: P value is significant.

Table S10. 2x2 contingency table for the association between unhealthy metabolic profiles and unhealthy gut microbiome profiles

a. Generation R Study

	**Cluster results based on gut microbiome profile**		
**Cluster results based on metabolic profile**	Cluster H	Cluster U	Total
Metabolically healthy	545	417	962
Metabolically unhealthy	261	265	526
Total	806	682	1488

b. Rotterdam Study

	**Cluster results-based gut microbiome profile**		
**Cluster results based metabolic profile**	Cluster H	Cluster U	Total
Metabolically healthy	345 (H-H)	329 (H-U)	674
Metabolically unhealthy	233 (U-H)	358 (U-U)	591
Total	578	687	1265

c. Lifelines-DEEP Study

	**Cluster results based on gut microbiome profile**		
**Cluster results based on metabolic profile**	Cluster H	Cluster U	Total
Metabolically healthy	253	277	530
Metabolically unhealthy	177	222	399
Total	430	499	929

Table S11. Risk of atherosclerotic cardiovascular disease (ASCVD) between two microbiome clusters

**Model**#	**HR (95% CI)***	**P value**
**Model 1**	1·59 (0·88-2·87)	0·13
**Model 2**	1·63 (0·90-2·95)	0·11
**Model 3**	1·50 (0·82-2·73)	0·19
**Model 4**	1·52 (0·83-2·80)	0·17

"*": microbiome cluster H as reference

"#":
Model 1: unadjusted model;
Model 2: adjust for TimeInMail, sampling season, DNA isolation batch, and sequencing batch;
Model 3: Model 2 additionally adjusted for age, sex, BMI;
Model 4: Model 3 additionally adjusted for smoking, alcohol intake, education level, and lipids lowering medication use.

Table S12. Univariate comparison of metabolic clusters classified by K-Means clustering based on host metabolic phenotypes within Rotterdam Study, Generation R Study and Lifelines-DEEP Study.

	**Generation R Study (N=1488)**			**Rotterdam Study (N=1265)**			**Lifelines-DEEP Study (N=929)**		
	**Cluster H (n= 962)**	**Cluster U (n= 526)**	**P value**	**Cluster H (n=674)**	**Cluster U (n=591)**	**P value**	**Cluster H (n= 530)**	**Cluster U (n= 399)**	**P value**
Age (years)	9·80 ± 0·30	9·90 ± 0·40	< 0·001 ***	61·90 ± 5·50	63·60 ± 5·80	< 0·001 ***	39·40 ± 12·00	52·50 ± 12·00	<0·001***
Sex (male,%)	54·90% (528)	43·30% (228)	< 0·001 ***	30·00% (202)	55·20% (326)	< 0·001 ***	25·00% (133)	62·20% (248)	<0·001***
BMI	16·00 ± 1·10	19·80 ± 2·40	< 0·001 ***	24·90 ± 2·90	30·00 ± 4·00	< 0·001 ***	23·30 ± 2·90	27·90 ± 3·90	<0·001***
Overweight prevalence	0·00% (0)	44·30% (233)	< 0·001 ***	42·10% (284)	48·90% (289)	0·02 *	20·20% (107)	52·90% (211)	<0·001***
Obesity prevalence	0·00% (0)	6·50% (34)	<0·001 ***	4·60% (31)	44·50% (263)	< 0·001 ***	2·10% (11)	25·60% (102)	<0·001***
Body fat (%)	25·90 ± 4·30	35·90 ± 5·10	< 0·001 ***	33·20 ± 7·20	37·80 ± 6·60	< 0·001 ***	NA	NA	NA
Waist-to-hip ratio	NA	NA	NA	0·80 ± 0·070	1·00 ± 0·09	< 0·001 ***	0·90 ± 0·07	1·00 ± 0·06	<0·001***
Triglycerides (log_{10}-) (mmol/L)	-0·10 ± 0·40	0·10 ± 0·50		0·030 ± 0·15	0·22 ± 0·19	< 0·001 ***	-0·11 ± 0·21	0·12 ± 0·17	<0·001***
HDL_c (mmol/L)	1·60 ± 0·30	1·40 ± 0·30	< 0·001 ***	1·70 ± 0·50	1·30 ± 0·30	< 0·001 ***	1·70 ± 0·40	1·40 ± 0·40	<0·001***

Cholesterol (mmol/L)	4·30 ± 0·60	4·40 ± 0·60	< 0·01 ***	5·80 ± 1·10	5·30 ±1·10	< 0·001 ***	4·70 ± 0·90	5·40 ± 1·00	<0·001***
Glucose (mmol/L)	5·20 ± 1·00	5·40 ± 0·90	0·01**	5·30 ± 0·50	6·20 ±1·30	< 0·001 ***	4·70 ± 0·90	5·30 ± 0·90	<0·001***
Insulin (log-) (pmol/L)	5·00 ± 0·70	5·40 ± 0·70	< 0·001 ***	1.73 ± 0.18	2.02 ± 0·24	< 0·001 ***	NA	NA	NA
Insulin resistance	NA	NA	NA	2·00 ± 0·90	5·00 ± 4·80	< 0·001 ***	NA	NA	NA
T2D prevalence	NA	NA	NA	3·00% (20)	22·00% (130)	< 0·001***	0·00% (0)	3·00% (12)	<0·001***
Mean SBP (mmHg)	100·20 ± 6·90	107·30 ± 7·20	< 0·001 ***	127·30 ± 16·50	142·10 ± 17·20	< 0·001 ***	112·20 ± 9·00	128·30 ± 12·40	<0·001***
Mean DBP (mmHg)	57·00 ± 6·20	60·60 ± 6·40	< 0·001 ***	77·70 ± 9·60	86·50 ± 10·30	< 0·001 ***	66·50 ± 6·90	76·50 ± 8·70	<0·001***
Hypertension prevalence	NA	NA	NA	38·70% (261)	81·10% (479)	< 0·001 ***	9·90% (51)	33·30% (127)	<0·001***

CRP									
-- <1 mg/L	89·50% (861)	67·50% (355)					55·10% (292)	30·90% (123)	
-- 1~3 mg/L	8·30% (80)	20·90% (110)	<0·001 ***	NA	NA	NA	28·10% (149)	45·50% (181)	<0·001***
-- >3 mg/L	2·20% (21)	11·60% (61)					16·80% (89)	23·60% (94)	

"*": p<0·05; "**": p<0·01; "***": p<0·001; "NA": Data not available

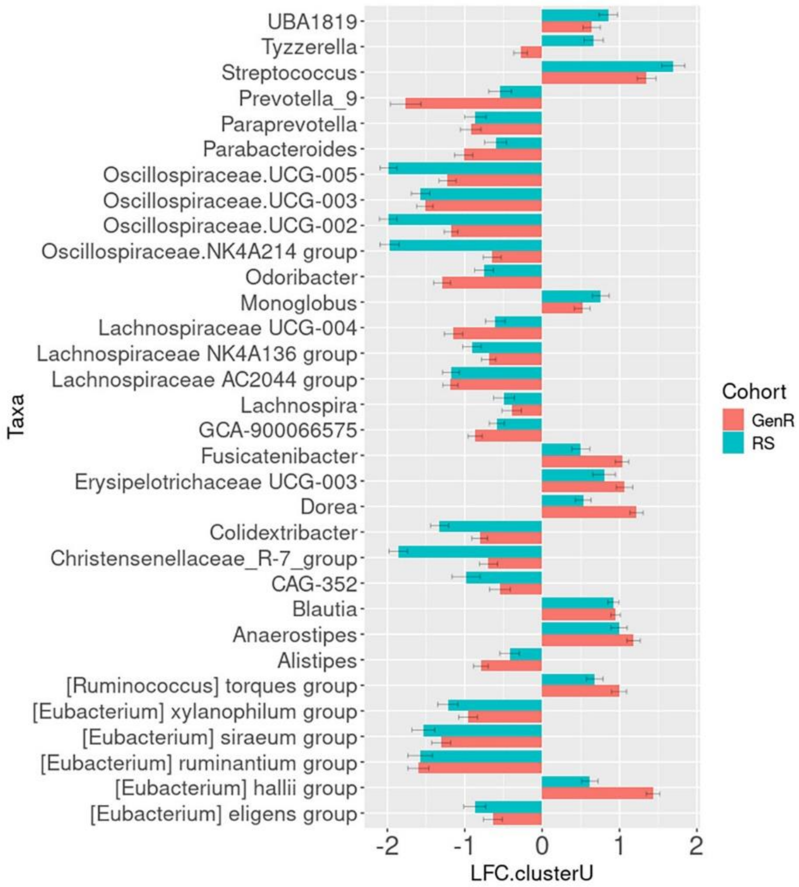

Figure S1. Overlapped cluster-driving genera between Rotterdam Study and Generation R Study. Bars represent log-fold change in genera abundance for cluster U (cluster H used as reference). Red bars refer to Generation R Study, and green bars refer to Rotterdam Study. “LFC.clusterU”=Log-fold change for cluster U compared to cluster H.

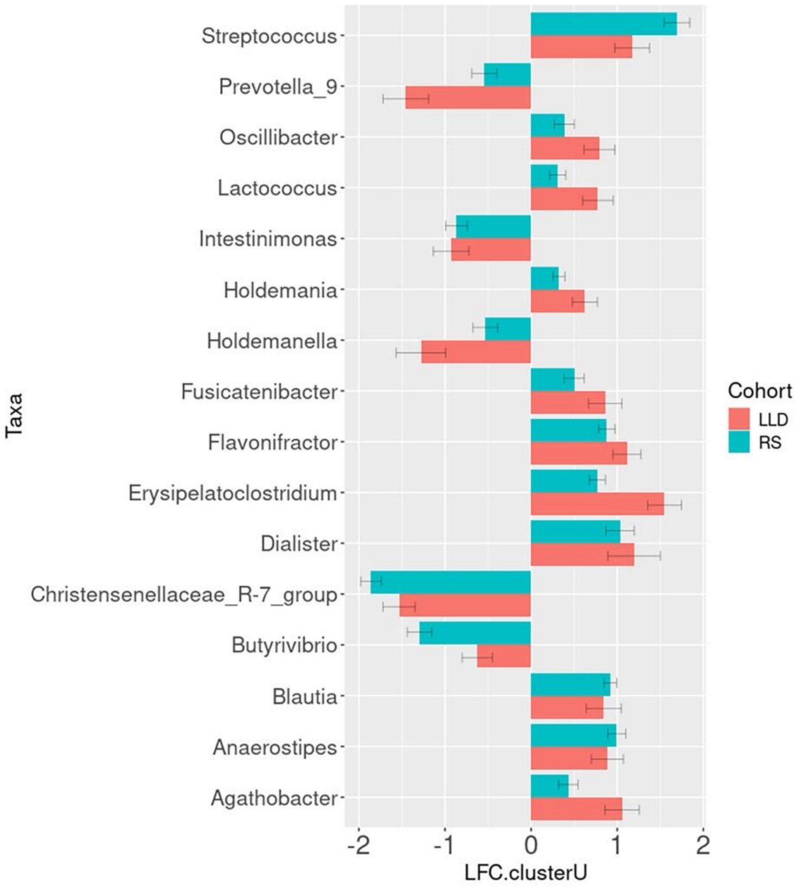

Figure S2. Overlapped cluster-driving genera between Rotterdam Study and Lifelines-DEEP Study. Bars represent log-fold change in genera abundance for cluster U (cluster H used as reference). Red bars refer to Lifelines-DEEP Study, and green bars refer to Rotterdam Study. “LFC.clusterU”=Log-fold change for cluster U compared to cluster H.

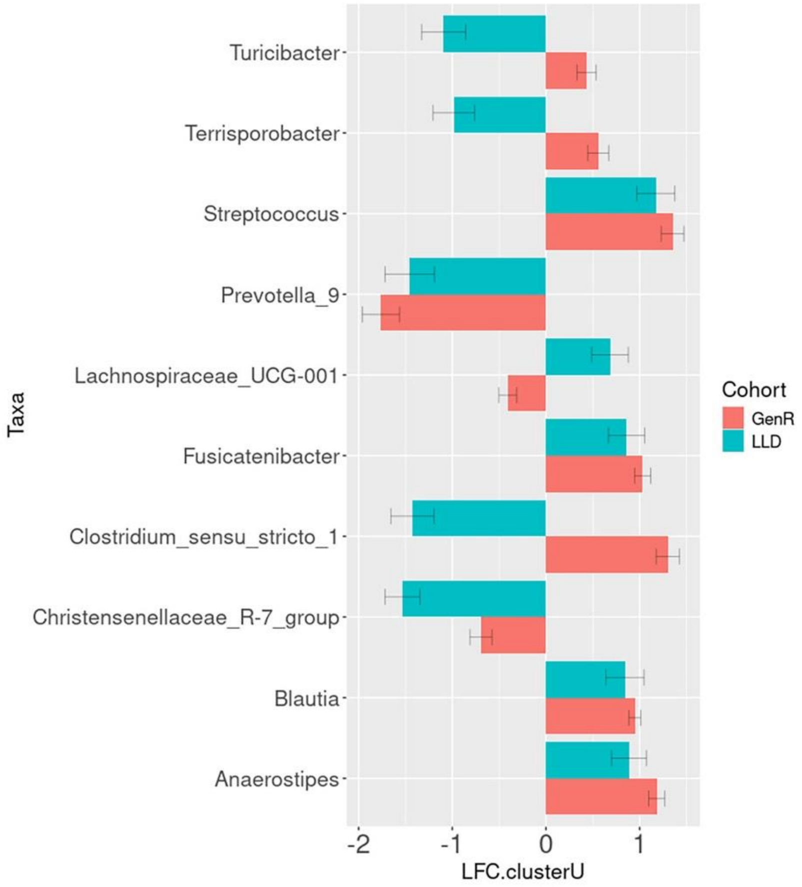

Figure S3. Overlapped cluster-driving genera between Generation R Study and Lifelines-DEEP Study. Bars represent log-fold change in genera abundance for cluster U (cluster H used as reference). Red bars refer to Generation R Study, and green bars refer to Lifelines-DEEP Study. “LFC.clusterU”=Log-fold change for cluster U compared to cluster H.

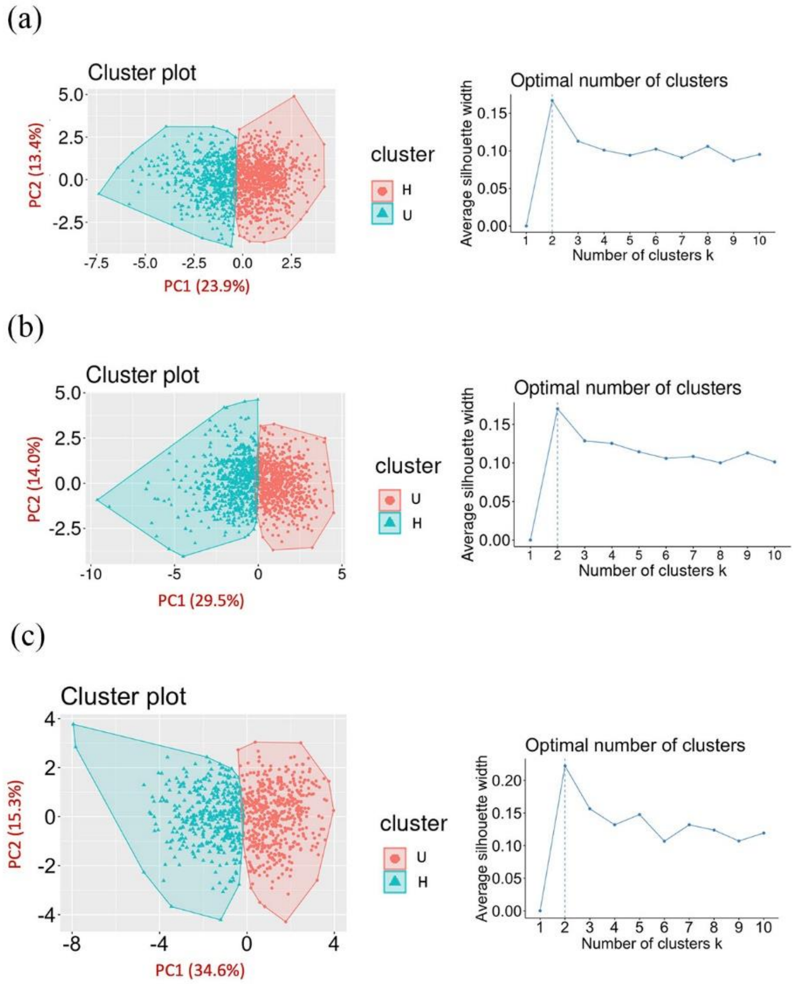

Figure S4. Two-dimensional representation of the metabolic phenotypes-based clustering of (a) Generation R Study, (b) Rotterdam Study, and (c) Lifelines-DEEP Study. Visualization of first two principal components of samples labeled with cluster assignment by K-means of the corresponding metabolic phenotypes. The results of the average silhouette width algorithm to select the optimal number of clusters are shown in the right panels.

References

1. Radjabzadeh D, Boer CG, Beth SA, et al. Diversity, compositional and functional differences between gut microbiota of children and adults. *Scientific Reports* 2020; **10**(1): 1040.
2. Chen Z, Radjabzadeh D, Chen L, et al. Association of Insulin Resistance and Type 2 Diabetes With Gut Microbial Diversity: A Microbiome-Wide Analysis From Population Studies. *JAMA Netw Open* 2021; **4**(7): e2118811.
3. Schmieder R, Lim YW, Rohwer F, Edwards R. TagCleaner: Identification and removal of tag sequences from genomic and metagenomic datasets. *BMC Bioinformatics* 2010; **11**(1): 341.
4. Callahan BJ, McMurdie PJ, Rosen MJ, Han AW, Johnson AJ, Holmes SP. DADA2: High-resolution sample inference from Illumina amplicon data. *Nat Methods* 2016; **13**(7): 581-3.
5. Wang Q, Garrity GM, Tiedje JM, Cole JR. Naive Bayesian classifier for rapid assignment of rRNA sequences into the new bacterial taxonomy. *Appl Environ Microbiol* 2007; **73**(16): 5261-7.
6. Quast C, Pruesse E, Yilmaz P, et al. The SILVA ribosomal RNA gene database project: improved data processing and web-based tools. *Nucleic Acids Res* 2013; **41**(Database issue): D590-6.
7. Kurilshikov A, Medina-Gomez C, Bacigalupe R, et al. Large-scale association analyses identify host factors influencing human gut microbiome composition. *Nature Genetics* 2021; **53**(2): 156-65.
8. Cole TJ, Lobstein T. Extended international (IOTF) body mass index cut-offs for thinness, overweight and obesity. *Pediatr Obes* 2012; **7**(4): 284-94.
9. WHO. Obesity. https://wwwwhoint/health-topics/obesity#tab=tab_1 Accessed on July 12, 2023.
10. Alferink LJM, Trajanoska K, Erler NS, et al. Nonalcoholic Fatty Liver Disease in The Rotterdam Study: About Muscle Mass, Sarcopenia, Fat Mass, and Fat Distribution. *J Bone Miner Res* 2019; **34**(7): 1254-63.
11. Jaddoe VWV, Bakker R, van Duijn CM, et al. The Generation R Study Biobank: a resource for epidemiological studies in children and their parents. *European Journal of Epidemiology* 2007; **22**: 917 - 23.
12. Matthews DR, Hosker JP, Rudenski AS, Naylor BA, Treacher DF, Turner RC. Homeostasis model assessment: insulin resistance and β-cell function from fasting plasma glucose and insulin concentrations in man. *Diabetologia* 1985; **28**(7): 412-9.
13. Monasso GS, Jaddoe VWV, Küpers LK, Felix JF. Epigenetic age acceleration and cardiovascular outcomes in school-age children: The Generation R Study. *Clinical Epigenetics* 2021; **13**(1): 205.
14. WHO. World Health Organization Anatomical Therapeutic Chemical (WHO ATC) classification [Internet]. https://wwwwhoccno/atc_ddd_index/ Last update Dec 2017.
15. Brahimaj A, Ligthart S, Ikram MA, et al. Serum Levels of Apolipoproteins and Incident Type 2 Diabetes: A Prospective Cohort Study. *Diabetes Care* 2016; **40**(3): 346-51.
16. Leening MJG, Kavousi M, Heeringa J, et al. Methods of data collection and definitions of cardiac outcomes in the Rotterdam Study. *European Journal of Epidemiology* 2012; **27**(3): 173-85.
17. Netherlands S. Allochtonen in Nederland 2004. *Voorburg/Heerlen* 2004.
18. Medina-Gomez C, Heppe DHM, Yin JL, et al. Bone Mass and Strength in School-Age Children Exhibit Sexual Dimorphism Related to Differences in Lean Mass: The Generation R Study. *J Bone Miner Res* 2016; **31**(5): 1099-106.

19. Szekely E, Neumann A, Sallis H, et al. Maternal Prenatal Mood, Pregnancy-Specific Worries, and Early Child Psychopathology: Findings From the DREAM BIG Consortium. *Journal of the American Academy of Child & Adolescent Psychiatry* 2021; **60**(1): 186-97.
20. Chung WK, De Vos-Jakobs S, Rivadeneira F, Bierma-Zeinstra SM, Waarsing JH. The association of BMI and physical activity on acetabular dysplasia in children. *Osteoarthritis and Cartilage* 2021; **29**(1): 50-8.
21. van der Velde LA, Nguyen AN, Schoufour JD, et al. Diet quality in childhood: the Generation R Study. *Eur J Nutr* 2019; **58**(3): 1259-69.
22. Chen Z, Schoufour JD, Rivadeneira F, et al. Plant-based Diet and Adiposity Over Time in a Middle-aged and Elderly Population: The Rotterdam Study. *Epidemiology* 2019; **30**(2): 303-10.
23. Waqas K, Chen J, van der Eerden BCJ, et al. Dietary Advanced Glycation End-Products (dAGEs) Intake and Bone Health: A Cross-Sectional Analysis in the Rotterdam Study. *Nutrients* 2020; **12**(8): 2377.
24. Chen Z, Zuurmond MG, van der Schaft N, et al. Plant versus animal based diets and insulin resistance, prediabetes and type 2 diabetes: the Rotterdam Study. *Eur J Epidemiol* 2018; **33**(9): 883-93.
25. Wendel-Vos GC, Schuit AJ, Saris WH, Kromhout D. Reproducibility and relative validity of the short questionnaire to assess health-enhancing physical activity. *J Clin Epidemiol* 2003; **56**(12): 1163-9.
26. Scholtens S, Smidt N, Swertz MA, et al. Cohort Profile: LifeLines, a three-generation cohort study and biobank. *Int J Epidemiol* 2015; **44**(4): 1172-80.
27. Gloor GB, Macklaim JM, Pawlowsky-Glahn V, Egozcue JJ. Microbiome Datasets Are Compositional: And This Is Not Optional. *Front Microbiol* 2017; **8**: 2224.
28. Lin H, Peddada SD. Analysis of compositions of microbiomes with bias correction. *Nature Communications* 2020; **11**(1): 3514.
29. McMurdie PJ, Holmes S. phyloseq: An R Package for Reproducible Interactive Analysis and Graphics of Microbiome Census Data. *PLOS ONE* 2013; **8**(4): e61217.
